# SpatConv Enables the Accurate Prediction of Protein Binding Sites by a Pretrained Protein Language Model and an Interpretable Bio-spatial Convolution

**DOI:** 10.34133/research.0773

**Published:** 2025-07-08

**Authors:** Mingming Guan, Jiyun Han, Shizhuo Zhang, Hongyu Zheng, Juntao Liu

**Affiliations:** ^1^ School of Mathematics and Statistics, Shandong University, Weihai 264209, China.; ^2^ Department of Radiation Oncology, Qilu Hospital, Cheeloo College of Medicine, Shandong University, Jinan 250012, China.

## Abstract

Protein interactions with molecules, such as other proteins, peptides, or small ligands, play a critical role in biological processes, and the identification of protein binding sites is crucial for understanding the mechanisms underlying diseases such as cancer. Traditional protein binding site prediction models usually extract residue features manually and then employ a graph or point-cloud-based architecture borrowed from other fields. Therefore, substantial information loss and limited learning ability cause them to fail to capture residue binding patterns. To solve these challenges, we introduce a general network that predicts the binding residues of proteins, peptides, and metal ions on proteins. SpatConv extracts sequence features from a pretrained large protein language model and structure features from a local coordinate framework. SpatConv learns residue binding patterns through a specially designed, graph-free bio-spatial convolution, which characterizes the complex spatial environments around the residues. After training and testing, SpatConv demonstrates great improvements over the state-of-the-art predictors and reveals novel biological insights into the relationship between binding sites and physicochemical properties. Notably, SpatConv exhibits robust performance across predicted and experimental structures, enhancing its reliability. Additionally, when applying it to the spike protein structure of severe acute respiratory syndrome coronavirus 2, SpatConv successfully identifies antibody binding sites and predicts potential binding regions, providing strong evidence supporting new drug development. A user-friendly online server for SpatConv is freely available at http://liulab.top/SpatConv/server.

## Introduction

Protein interactions with molecules like peptides, proteins, and metal ions are critical for cellular functions, influencing processes like gene expression and metabolism. Disruptions in these interactions are linked to diseases such as diabetes and cardiovascular disorders [1–6]. Therefore, accurate identification of protein binding sites is crucial for understanding disease mechanisms and developing therapeutic strategies [7]. However, predicting protein binding sites is challenging due to the complexity of protein structures and the intricate nature of these interactions.

A comprehensive understanding of binding site mechanisms requires identifying specific residues facilitating protein interactions with molecules. Experimental techniques like immunoprecipitation [8], yeast 2-hybrid systems [9], and surface plasmon resonance [10] can delineate interaction sites but are costly and time-consuming. As a result, computational methods for binding site prediction have become essential [11,12]. These methods are generally divided into sequence-based and structure-based approaches. Sequence-based methods, such as iPPBS-Opt [13], PepBind [14], PepBCL [15], and TargetS [16], analyze amino acid sequences, benefiting from the vast availability of sequence data, but often lack crucial tertiary structure information. Structure-based methods, such as GraphBind [17], IonCom [18], and ScanNet [19], leverage geometric feature extraction and structural context analysis to achieve enhanced predictive accuracy for protein–protein, peptide, and metal ion interactions, albeit at the cost of substantial computational requirements.

Many existing methods rely on evolutionary features derived from multiple sequence alignments, such as position-specific scoring matrices and hidden Markov models, which are computationally expensive and limit scalability [20]. In contrast, recent advances in protein language models (PLMs) offer an efficient alternative by learning contextual residue representations directly from sequences without requiring multiple sequence alignments. These pretrained models have already demonstrated strong performance in binding site prediction and other structural biology tasks [21–24], providing a fast and effective feature extraction pipeline.

Existing methods for protein binding site prediction often rely on general-purpose deep learning models, such as convolutional neural networks [25], graph neural networks (GNNs) [26], and transformers [27], to aggregate local neighborhood information. However, these approaches generally lack explicit modeling of the geometric orientation of local protein structures. For instance, convolutional neural networks are inherently designed for regular grid data and are ill-suited for irregular 3-dimensional (3D) molecular geometries. While transformers are powerful in capturing global dependencies, they struggle to encode spatial relationships in local 3D environments. Conventional GNNs, despite their efficacy on graph-structured data, often overlook spatial directionality and are typically invariant to rotations and translations, which limits their ability to learn fine-grained spatial patterns critical in protein structures. Although SE(3)-equivariant GNNs have demonstrated strong capability in modeling molecular symmetries [28], their high computational cost and architectural complexity make them less practical for large-scale biomolecular analysis.

Specialized methods like GeoBind [29] leverage local reference frames to predict nucleic binding sites by aggregating neighboring point cloud information on protein surfaces, while ScanNet builds atom and amino acid representations based on their spatio-chemical arrangement in 3D space. GraphBind, on the other hand, constructs hierarchical graphs from the structural context of target residues and their neighbors to capture local patterns for nucleic acid binding prediction. Despite their advancements, these methods still rely on graph-based representations, which face challenges such as limited resolution, flexibility, and computational inefficiency when applied to large-scale biomolecular analysis.

To overcome the limitations of existing protein binding site predictors, we propose SpatConv, a novel geometric deep learning framework tailored for accurate and robust identification of binding sites. SpatConv introduces a graph-free architecture that overcomes the limitations of traditional graph-based models, specifically addressing the challenges of limited resolution and flexibility in capturing local residue contact patterns in large biomolecules. Firstly, unlike conventional models that rely on discrete graph topologies, SpatConv directly encodes local spatial context in a continuous and dense manner, enabling adaptive and fine-grained modeling of the geometric distributions and directional structural patterns of surrounding residues. This graph-free design is not constrained by edge definitions, greatly improving spatial resolution, computational efficiency, and robustness to structural noise, thus allowing for more accurate identification of binding residues. Secondly, SpatConv introduces an innovative fusion mechanism specifically designed to address the challenge of effectively fusing sequence and structural information in large-scale binding site prediction. This problem-driven fusion approach enables the model to more precisely capture the spatial–functional correlations of residue contact patterns in large biomolecules, largely enhancing the identification of binding sites.

Compared to traditional methods, SpatConv not only achieves synergistic modeling of sequence conservation and structural arrangement but also improves tolerance to low-quality predicted structures, highlighting its innovation and practicality in complex binding scenarios. Comprehensive evaluations on multiple challenging datasets demonstrate that SpatConv consistently outperforms leading sequence-based and structure-based predictors across 4 distinct tasks, including protein–protein, protein–peptide, and metal ion (Zn^2+^/Mn^2+^) interactions. Interpretability analysis further reveals that conventional physicochemical properties like solvent-accessible surface area (SASA) and hydrophobicity show limited correlation with binding site discrimination. SpatConv’s generalizable, lightweight, and interpretable architecture supports fine-grained biomolecular recognition and proves effective in real-world applications—for instance, accurately predicting antibody binding epitopes on the severe acute respiratory syndrome coronavirus 2 (SARS-CoV-2) spike protein, identifying potential nanobody and drug targets consistent with the existing literature, and providing valuable insights into antigenic properties and immune interactions that could accelerate vaccine and therapeutic development.

## Results

### Overview of the SpatConv framework

**Fig. 1.**
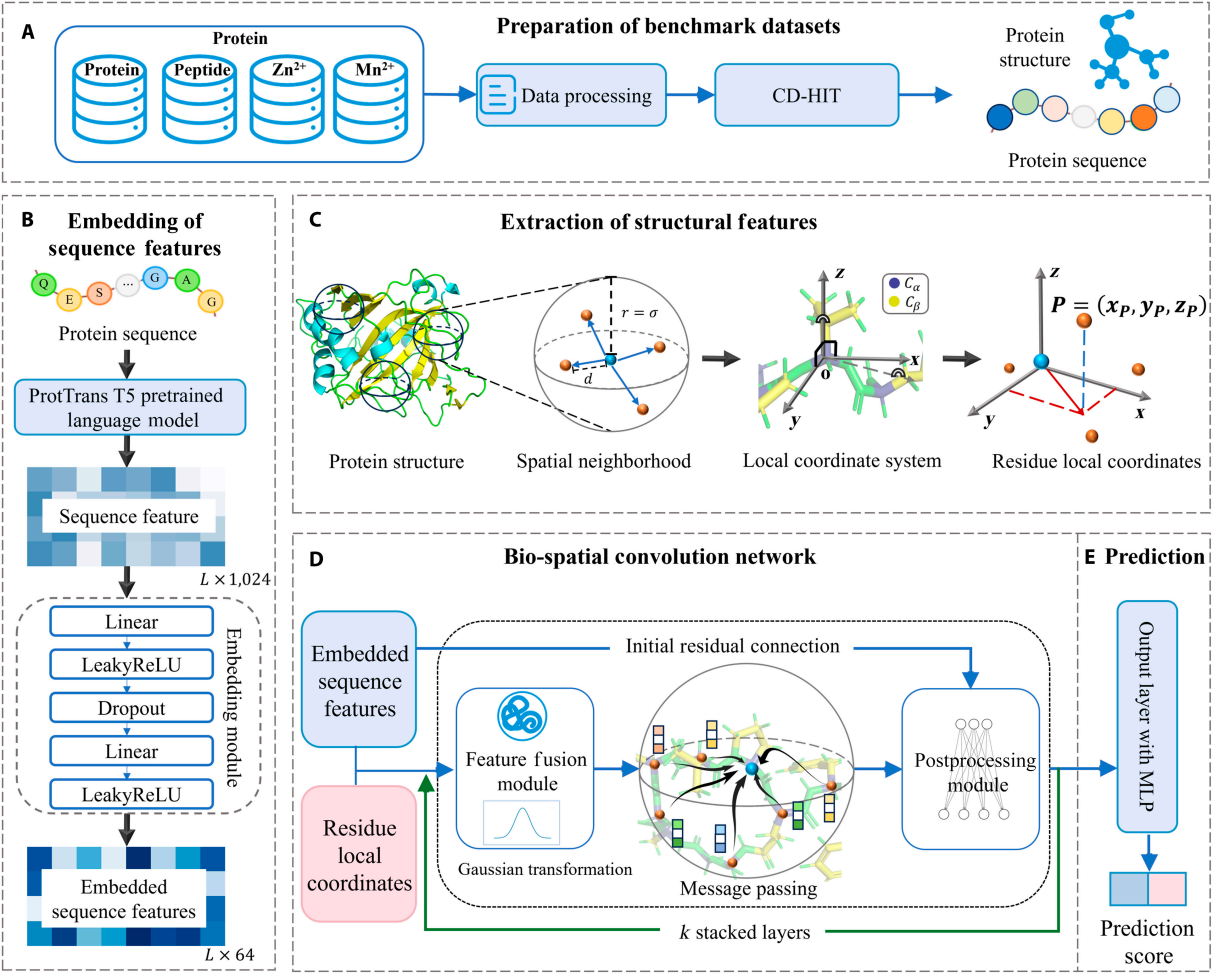
Overview of the SpatConv architecture. (A) Preparation of benchmark datasets. (B) Embedding of sequence features. The sequence is input into ProtT5 to generate a high-dimensional representation for each amino acid, which is then embedded by the embedding module. (C) Extraction of structural features. A local sphere is constructed centered on each residue, accompanied by a local coordinate system that maintains SE(3) equivariance. The neighboring residues’ spatial projections are computed within this coordinate system. (D) Bio-spatial convolution network. The fused sequence features and residue local coordinates are passed through *k* iterations of bio-spatial convolutions for learning the residual representations. (E) Prediction. A multilayer perceptron is used to predict the residue binding probabilities. MLP, multilayer perceptron.

SpatConv takes the protein sequence and structure as inputs and outputs a residue-wise label probability. The overall framework of SpatConv, as shown in Fig. 1 and detailed in Methods, consists of 5 main components: (a) preparation of benchmark datasets, (b) extraction of sequence features for each residue, (c) extraction of structural features for each residue, (d) iterative learning of residue representations through the bio-spatial convolution encoder, and (e) generation of residue prediction probabilities.

To support a wide range of biomolecular interactions—including protein–protein, protein–peptide, and metal ion (Zn^2+^/Mn^2+^) binding—SpatConv preprocesses protein data to obtain both refined sequences and structure inputs (Fig. 1A). It begins by using the pretrained ProtT5 PLM to extract high-dimensional semantic embeddings that capture rich residue-level biochemical and evolutionary information (Fig. 1B). SpatConv then constructs residue-centric local coordinate systems that are invariant to global rotation and translation. Instead of building an explicit graph, it projects neighboring residues into this local frame to compute their spatial distribution as continuous, dense geometric descriptors (Fig. 1C). These descriptors encode the precise 3D positions and directional relationships of surrounding residues in real-valued space, capturing subtle structural patterns that might be lost in sparse, discrete graph-based representations. By embedding neighboring residues in a residue-centered local frame, the model can more accurately perceive how residues are spatially arranged around a target residue—capturing subtle geometric patterns and directional cues essential for identifying binding sites. This approach not only enhances spatial sensitivity but also results in a more flexible and efficient model architecture.

To effectively fuse both sequence and structural information, SpatConv employs a Feature Fusion Module that aligns semantic embeddings with their geometric context, learning cross-modal dependencies between sequence signals and spatial environments. This fusion mechanism enables the model to reason jointly about conserved biochemical patterns and their structural manifestations—providing a more holistic and biologically meaningful representation of each residue. In the final learning phase (Fig. 1D), SpatConv applies a series of bio-spatial convolutions with Gaussian distance-based weighting to iteratively refine the fused features. Through iterative bio-spatial convolutions, the model progressively refines its understanding of the local chemical environment, ultimately assigning a binding likelihood score to each residue (Fig. 1E).

### SpatConv shows great superiority over other state-of-the-art methods

In this section, we evaluate the performance of SpatConv against several other the state-of-the-art methods including GraphPPIS [30], MaSIF-site [31], ScanNet, PeSTo [32], and Spatom [33] evaluated on protein binding test sets; PepBind, PepNN [34], and PepBCL evaluated on peptide binding test sets; MIB [35], TargetS, and IonCom evaluated on Zn^2+^ binding test sets; and MIB, TargetS, IonCom, and GraphBind evaluated on Mn^2+^ binding test sets. We collected the 4 binding site datasets based on protein structures from the Protein Data Bank (PDB) [36], sourced from multiple databases as detailed in Methods. In this study, recall, precision, F1 score, Matthews correlation coefficient (MCC) [37], area under the receiver operating characteristic curve (AUROC) [38], and area under the precision–recall curve (AUPRC) [39] are applied as evaluation metrics (formulas in Note S1). Given the trade-off between recall and precision, we primarily adopt F1 score, MCC, AUROC, and AUPRC for a comprehensive performance assessment.

**Fig. 2.**
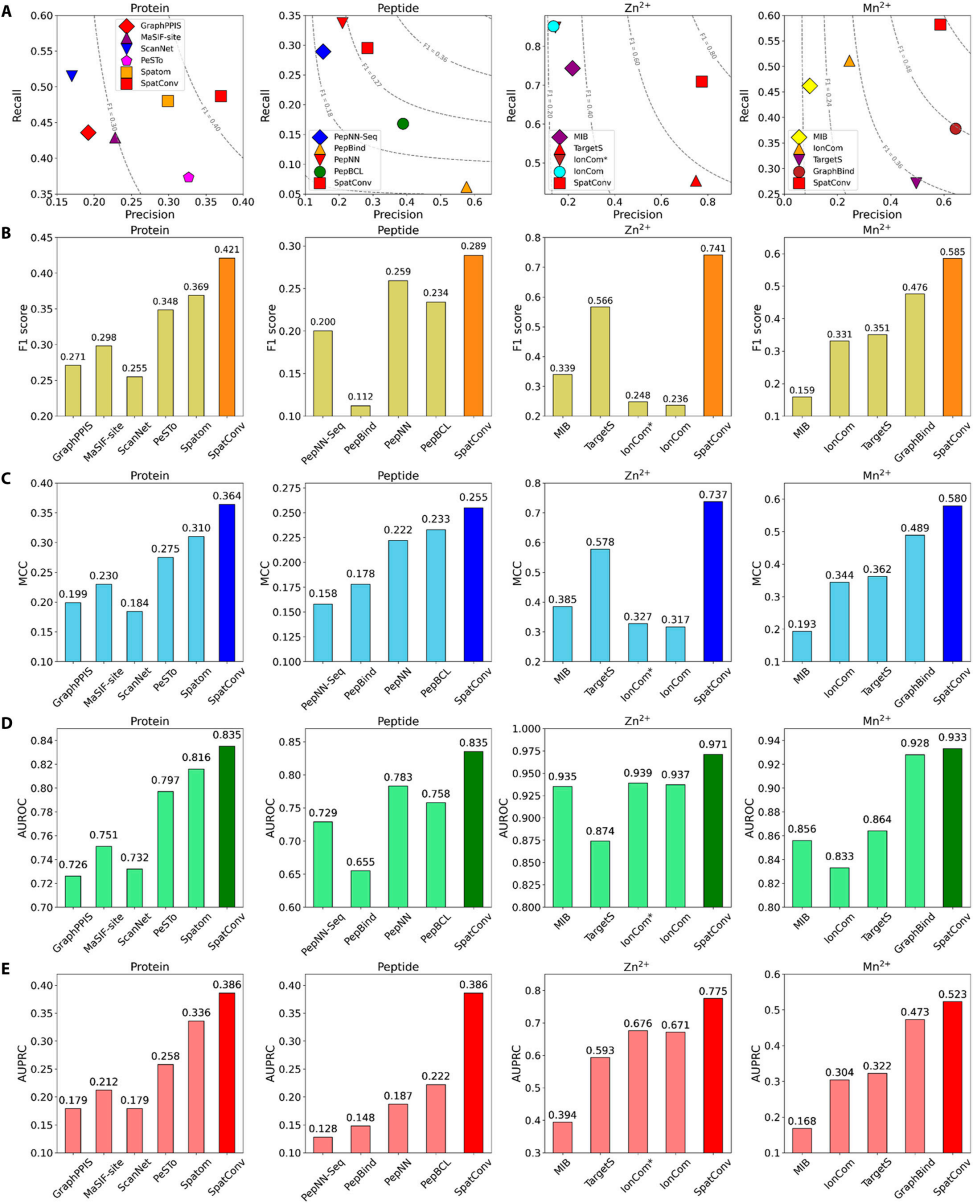
Performance comparison between SpatConv and other state-of-the-art predictors. Performance is evaluated across protein, peptide, Zn^2+^, and Mn^2+^ binding site prediction tasks using the (A) precision and recall, (B) F1 score, (C) Matthews correlation coefficient (MCC), (D) area under the receiver operating characteristic curve (AUROC), and (E) area under the precision–recall curve (AUPRC) metrics. Methods marked with an asterisk * were evaluated using ESMFold-predicted structures as input.

The results of the performance comparison are presented in Fig. 2 and Table S1. On the protein–protein binding site test set, SpatConv achieves superior performance with an F1 score of 0.421, an MCC of 0.364, an AUROC of 0.835, and an AUPRC of 0.386. Notably, SpatConv outperforms the second best method by 14.1% in F1 score, 17.4% in MCC, 2.3% in AUROC, and 14.9% in AUPRC (see Fig. 2A to E). SpatConv outperforms ScanNet, MaSIF, and PeSTo mainly due to its integration of pretrained features and bio-spatial convolution. Additionally, compared to our previously developed tool, Spatom, SpatConv eliminates the dependency on predefined graph structures, capturing residue binding patterns more accurately and greatly enhancing its performance. SpatConv’s superiority can be attributed to its integration of pretrained features and bio-spatial convolution, which effectively combine local and global structural information to enhance prediction accuracy. Moreover, after a comparison on an additional dataset based on a revised temporal split, SpatConv consistently demonstrates superior performance across all evaluation metrics (see Fig. S22 and Table S10), which highlights SpatConv’s strong generalization ability to newly discovered binding sites. The detailed temporal split methodology is described in the Methods section.

On the peptide binding site test set, SpatConv outperforms PepBCL, a contrastive learning framework for peptide binding site prediction using protein sequences, with an F1 score of 0.295 (26.1% improvement), an MCC of 0.261 (12.0% improvement), an AUROC of 0.785 (3.6% improvement), and an AUPRC of 0.241 (8.6% improvement). Among structure-based methods, PepNN, which incorporates structural features through an attention-based network, achieves an F1 score of 0.238. Sequence-based methods, however, struggle to capture complex binding patterns, as evidenced by PepNN-Seq and PepBind, with F1 scores of 0.200 and 0.112, respectively—translating to SpatConv improvements of 47.5% and 163.4%, respectively.

For the Zn^2+^ binding site test set, SpatConv delivers exceptional performance, as shown in Fig. 2 and Table S1, achieving an F1 score of 0.753, an MCC of 0.754, an AUROC of 0.972, and an AUPRC of 0.783. Compared to TargetS, the second best method in this dataset, SpatConv demonstrates a substantial improvement of 33.0% in F1 score. Other methods, such as IonCom, which integrates sequence-based information and structure-based features for metal ion binding site prediction, and MIB, a traditional metal ion binding prediction tool, record F1 scores of 0.236 and 0.339, respectively—corresponding to SpatConv improvements of 218.6% and 122.1%, respectively. This substantial performance gap highlights SpatConv’s ability to effectively capture the intricate binding patterns of Zn^2+^ ions.

On the Mn^2+^ binding site test set, SpatConv continues to excel, with an F1 score of 0.600, an MCC of 0.594, an AUROC of 0.953, and an AUPRC of 0.545. It surpasses GraphBind, a GNN-based approach, by 26.1% in F1 score. Other methods, including TargetS and IonCom, achieve F1 scores of 0.351 and 0.331, respectively, translating to SpatConv improvements of 70.9% and 81.3%, respectively, while MIB lags further behind with an F1 score of 0.159 (277.4% improvement by SpatConv). The consistent superiority of SpatConv across both Zn^2+^ and Mn^2+^ datasets underscores its robustness and adaptability in predicting metal ion binding sites, offering a powerful tool for understanding protein–metal interactions across diverse biological contexts.

The consistent superiority of SpatConv across protein–protein, peptide, and metal ion binding sites highlights its robustness and versatility in addressing diverse binding site prediction tasks. This success is largely due to its pretrained features, which overcome the limitations of manually extracting physicochemical properties, and its bio-spatial convolution encoder, which integrates local and global features for a comprehensive characterization of the residue environment.

We also included a training time comparison between SpatConv and the graph-based Spatom to validate the computational efficiency benefits of SpatConv’s graph-free design. This ensures a fair comparison as both methods use the same batch selection strategy. Specifically, Spatom requires 23 s per training epoch, while SpatConv completes the same task in 12 s. This large difference in training time directly reflects the advantages of SpatConv’s graph-free strategy. In terms of inference time, we recorded the inference times for multiple representative graph-based methods including Spatom, GraphBind, GraphPPIS, ScanNet, and MaSIF. Results showed that SpatConv again demonstrates great advantages, which lies not only in its graph-free design but also in its high efficiency of feature extraction strategy. All models were evaluated on the additional protein–protein binding dataset based on a revised temporal split and on the same platform (see Notes S4 and S5 for details).

### SpatConv is robust for different qualities of protein structures

A notable strength of SpatConv lies in its near-identical performance on predicted and experimental resolved structures, highlighting its robust tolerance to the qualities of protein structures. In this section, we provide a detailed robust analysis of SpatConv and explore its potential applications.

**Fig. 3.**
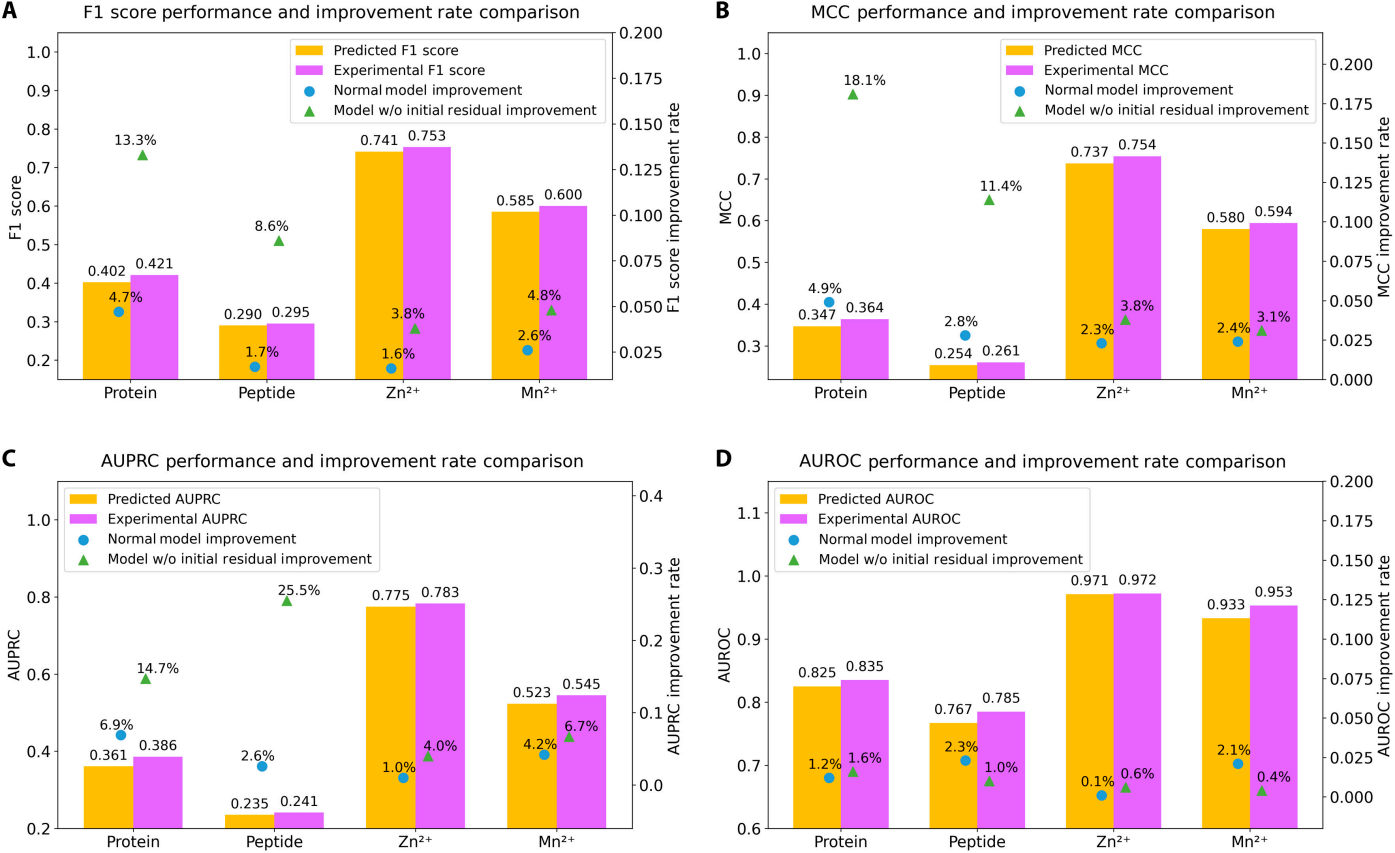
Performance and improvement rate comparison of SpatConv on predicted and experimental structures. This figure illustrates the performance evaluation and improvement rates of SpatConv across 4 datasets—protein, peptide, Zn^2+^, and Mn^2+^—using 4 metrics: (A) F1 score, (B) AUROC, (C) MCC, and (D) AUPRC. For each metric, the left *Y* axis shows the performance values for ESMFold-predicted structures (yellow bars) and experimental structures (purple bars), while the right *Y* axis displays the improvement rates of the corresponding metric, comparing the normal SpatConv model (light blue dots) and the model without initial residual connection (green triangles). The improvement rate is calculated as the relative difference in the corresponding metric between experimental and predicted structures, expressed as a percentage. The results highlight the impact of the initial residual connection on model robustness across diverse binding site prediction tasks.

We evaluated the robustness of SpatConv across 4 distinct datasets—protein, peptide, Zn^2+^, and Mn^2+^—using the F1 score, AUPRC, and AUROC metrics to compare its effectiveness on ESMFold-predicted and experimental structures. The results consistently demonstrate only slight differences between the 2 structure types, directly underscoring its strong robustness. As shown in Fig. 3A to D, the performance metrics are as follows: protein dataset: F1 score of 0.402 for predicted structures and 0.420 for experimental structures (difference: 4.29%); peptide dataset: F1 score of 0.290 for predicted structures and 0.295 for experimental structures (difference: 1.69%); Zn^2+^ dataset: F1 score of 0.741 for predicted structures and 0.753 for experimental structures (difference: 1.59%); and Mn^2+^ dataset: F1 score of 0.585 for predicted structures and 0.600 for experimental structures (difference: 2.50%). These results indicate that the performance of SpatConv on predicted structures is only slightly lower compared to that on experimental structures, further affirming the stability and reliability of SpatConv.

#### Sources of robustness and advantages

The robust performance of SpatConv on both predicted and experimental structures is primarily attributed to its incorporation of initial residual connections, which effectively integrate additional sequence information into the model. This design reduces SpatConv’s sensitivity to structural inaccuracies commonly found in predicted structures. Experimental analysis further supports this observation: when the initial residual connections are removed, the performance on predicted structures declines compared to that on experimental structures, with the performance gap becoming more pronounced, as illustrated in Fig. 3. For instance, in the protein dataset, the F1 score improvement rate increases from 4.7% in the normal model to 13.3% without initial residual connections, resulting in a performance gap of 8.6%. Similarly, for the peptide dataset, the F1 score improvement rate grows from 1.7% to 8.6% (a gap of 6.9%). For the Zn^2+^ dataset, the F1 score improvement rate rises from 1.6% to 3.8% (a gap of 2.2%), and for the Mn^2+^ dataset, it increases from 2.6% to 4.8% (a gap of 2.2%). These examples demonstrate that the absence of initial residual connections amplifies the performance disparity between predicted and experimental structures, highlighting the critical role of residual connections in enhancing robustness by leveraging sequence information to mitigate the impact of structural deviations. In contrast, many conventional methods [17,29] experience large performance declines when applied to predicted structures, underscoring SpatConv’s distinct advantage in handling diverse structural inputs with varying quality.

#### Implications for practical applications

SpatConv’s robustness provides key advantages for practical research as follows: Broadened applicability: Its reliability on predicted structures enables direct use for newly discovered or hard-to-crystallize proteins, bypassing the need for experimental data. Enhanced efficiency: By lessening reliance on high-quality experimental structures, SpatConv speeds up structural biology and drug development workflows, such as early-stage drug screening using predicted structures. Large-scale analysis: With tools like ESMFold generating vast predicted structure datasets, SpatConv’s accuracy makes it ideal for large-scale studies.

In summary, SpatConv’s near-identical performance on predicted and experimental structures highlights its technical strength and utility in structural biology. This reduces dependence on experimental data, enhancing its value for studying unknown proteins and drug design. SpatConv’s current robustness already demonstrates substantial practical benefits, with future studies potentially exploring complex scenarios such as multi-subunit or dynamic structures.

### Ablation studies on SpatConv

In this section, we present a series of ablation experiments to evaluate the impact of different components and conditions on the performance of SpatConv. First, we evaluate the contributions of nonsurface amino acids, structural information, and sequence–structure feature fusion by selectively removing or altering one component at a time. Then, we perform an ablation study on module replacement.

#### The contribution of nonsurface amino acids

**Fig. 4.**
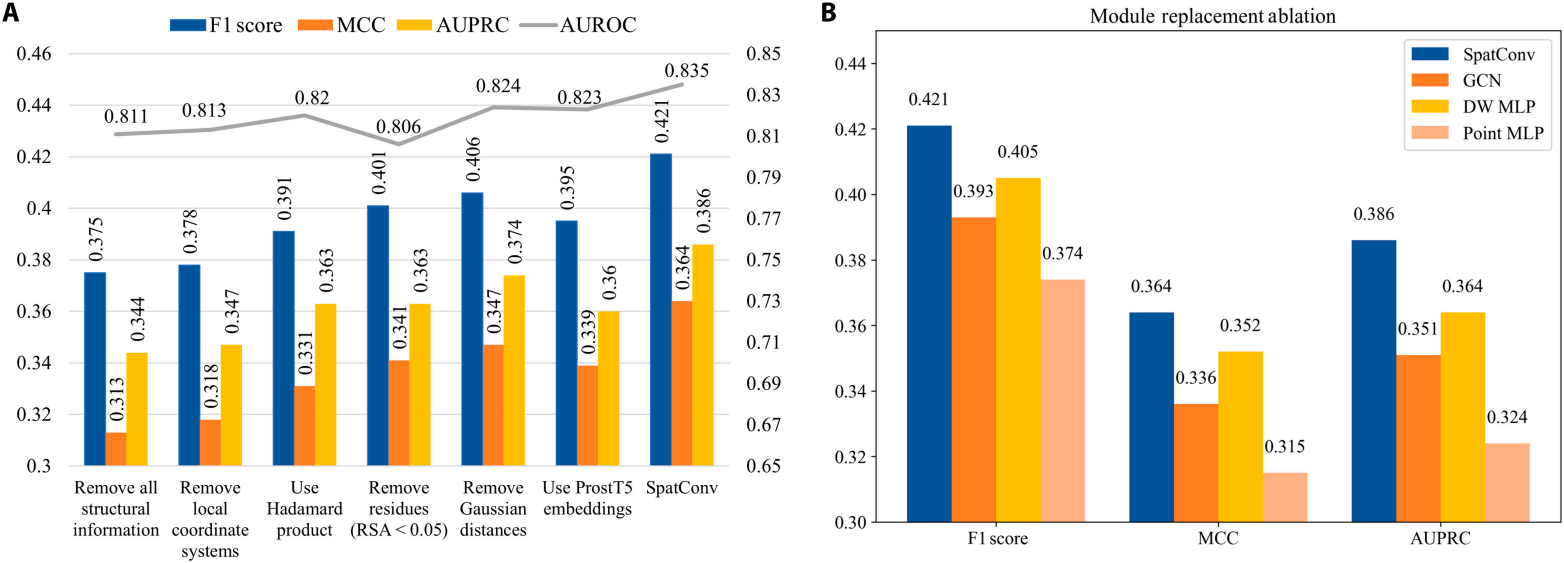
Performance comparison of ablation methods and module replacement ablation on the protein test set. (A) F1 score, MCC, and AUPRC are plotted on the primary axis (left axis). AUROC is plotted on the secondary axis (right axis). (B) Module replacement ablation study of the feature aggregation component. The proposed SpatConv is compared against 3 alternatives: graph convolutional network (GCN; graph-based), distance-weighted (DW) MLP (distance-aware but graph-free), and point MLP (fully graph-free). RSA, relative solvent accessibility.

While binding residues are typically located on the protein surface with high relative solvent accessibility (RSA) values, nonsurface residues (RSA < 0.05) are essential for the complete spatial topology of binding patterns. Methods such as Spatom, MaSIF, and GeoBind, which primarily focus on surface residues, fail to fully capture this topology by overlooking critical structural information from nonsurface regions. To test it, we trained and evaluated the model by excluding buried amino acids (RSA < 0.05). For protein–protein binding (Fig. 4A), the results showed a notable performance drop, with the F1 score decreasing by 4.75%, MCC by 6.32%, and other metrics similarly affected (reductions of 5.54% in recall, 4.05% in precision, 3.47% in AUPRC, and 5.96% in AUROC). This decline underscores the importance of buried residues in enhancing the learning ability of SpatConv in capturing spatial dependencies, emphasizing the necessity of including all residues. Similar results for the other 3 ligands are provided in Figs. S1 to S3.

#### The contribution of structural information

To evaluate the impact of structural information, we conducted 5 ablation experiments: (a) removing the local coordinate system, (b) removing the Gaussian distance, (c) eliminating all structural information, (d) replacing the feature fusion method with an element-wise multiplication (Hadamard product) of sequence and structural features, and (e) replacing sequence-based ProtT5 with structure-aware ProstT5 [40]. As shown in Fig. 4A, all modifications resulted in a clear performance degradation. Removing the local coordinate system has the most substantial impact on model performance, reducing the F1 score by 10.22% and the MCC by 12.68%. This underscores its crucial contribution in maintaining 3D spatial symmetry and capturing residue relationships. In contrast, eliminating the Gaussian distance yields a relatively mild impact (F1 score drop of 3.56%), suggesting its supplementary yet nonessential contribution to neighbor feature aggregation. Excluding all structural information severely impaired the model’s ability to correlate spatial conformation with binding sites, with the F1 score decreasing by 10.92%, affirming the pivotal role of structural information in prediction accuracy. Substituting the feature fusion with a Hadamard product diminished performance by 7.12% in F1 score, indicating its inferiority to our proposed method, which effectively integrates spatial and sequence information. Replacing ProtT5 with ProstT5, a structure-aware protein pretrained language model, led to a slight performance drop (F1 score drop of 6.18%). Overall, the local coordinate system and the full structural input emerge as the most critical components, contributing the most to the model’s predictive accuracy (see Tables S2 to S5 for details). Similar results for the other 3 ligands are provided in Figs. S1 to S3. These findings highlight the differential contributions of structural components, with the local coordinate system and comprehensive structural information emerging as particularly critical.

#### Module replacement ablation

To evaluate the effectiveness of the proposed graph-free convolution module (SpatConv), we conducted a module replacement ablation study on the task of protein binding site prediction. Under the same network architecture and training configuration, we systematically replaced the feature aggregation component with 3 representative alternatives as follows: (a) standard graph convolutional network: a conventional GNN model that explicitly constructs adjacency graphs, serving as a baseline for graph-based aggregation; (b) distance-weighted multilayer perceptron (MLP): a distance-aware but graph-free model that concatenates local spatial coordinates and residue features, weighted by pairwise distances, as input to an MLP for feature aggregation—representing a “weakly structured” alternative without explicit graph construction; and (c) point-based MLP (point MLP): A pure graph-free baseline that treats protein structures as point clouds, relying solely on local coordinate neighborhoods without any structural graph information. As shown in Fig. 4B, SpatConv consistently outperforms all alternative modules across multiple evaluation metrics. Compared to the graph convolutional network baseline, it achieves relative improvements of 7.1% in F1 score, 8.3% in MCC, and 10.0% in AUPRC. Instead of relying on an explicit graph structure, we select local neighbors for each residue based solely on spatial proximity—specifically, residues within a predefined radius—allowing our model to effectively capture local structural patterns while maintaining a lightweight, graph-free architecture. Against the distance-weighted MLP, SpatConv yields 4.0%, 3.4%, and 6.0% improvements, respectively, highlighting that merely incorporating spatial distance is less sufficient—our design introduces a more principled and protein-specific structural modeling strategy. When compared to the fully unstructured point MLP, SpatConv shows the most substantial gains, with 12.6%, 15.6%, and 19.1% improvements, demonstrating its superior ability to capture local geometric context while leveraging a streamlined, graph-free design. Supplementary binding data for the protein with the other 3 ligands are provided in Figs. S8 to S10, further validating the effectiveness of our approach.

### Interpretability analysis of SpatConv

To understand the mechanism of SpatConv in detecting binding/nonbinding sites, we conducted a comprehensive interpretability analysis from several aspects.

#### SpatConv learns characteristics related to binding residues

**Fig. 5.**
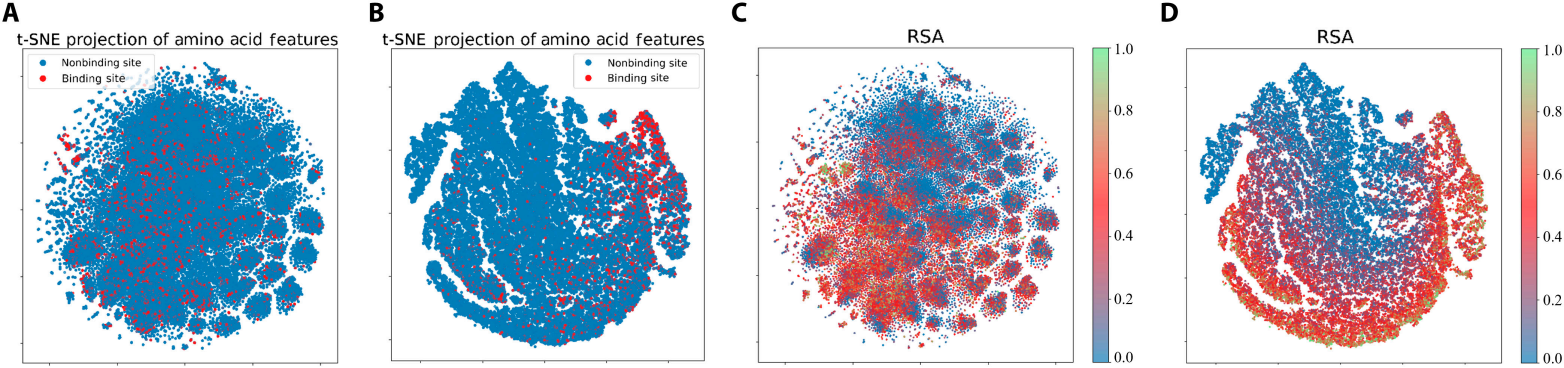
*t*-distributed stochastic neighbor embedding (t-SNE) projections of protein residue features before and after training. (A and B) t-SNE projections of raw and trained residue features for binding (red) and nonbinding (blue) sites. (C and D) t-SNE projections colored by RSA values before and after training.

We utilized *t*-distributed stochastic neighbor embedding (t-SNE) [41] visualization of residue features before and after training. The results for protein–protein binding are presented in Fig. 5, with supplementary binding data for the protein with the other 3 ligands provided in Figs. S11 to S13. Figure 5A presents the original residue features distribution, with no clear separation between binding and nonbinding sites. After training with SpatConv, Fig. 5B shows clear clustering of positive samples (binding sites), highlighting SpatConv’s ability to distinguish binding/nonbinding characteristics. In Fig. 5C, we visualize the distribution of original residue features colored by their RSA values. After training, Fig. 5D shows a distinct separation of RSA values, indicating that SpatConv successfully encodes RSA information, which is important for identifying binding sites [42]. The clustered binding sites in Fig. 5B align with regions of high RSA in Fig. 5D, confirming that binding residues are often located on protein surfaces with higher RSA values.

#### SpatConv’s shift in emphasis on physicochemical properties

**Fig. 6.**
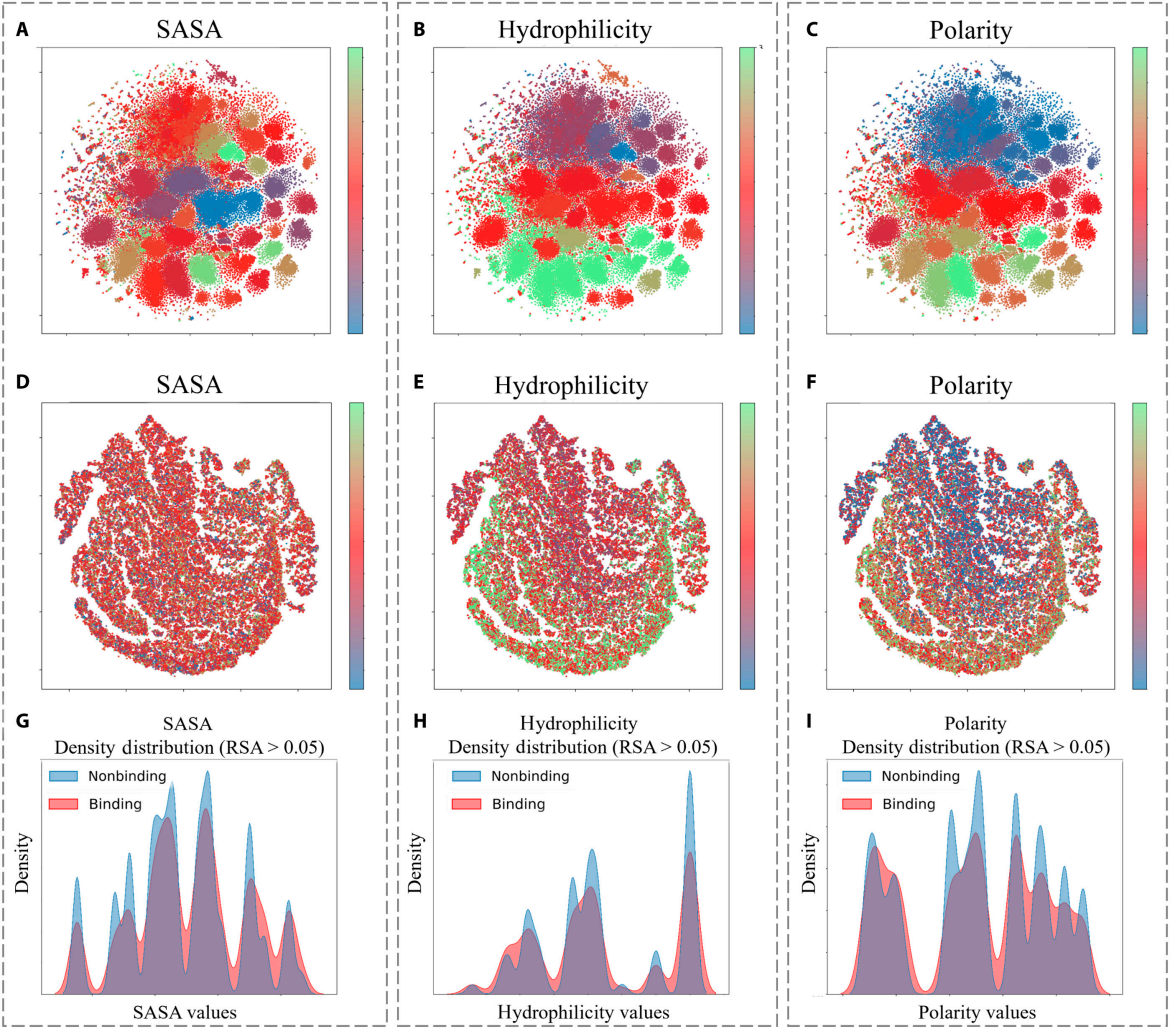
t-SNE visualization of protein physicochemical properties before and after SpatConv training, alongside density plots of physicochemical property distributions for binding/nonbinding sites. (A to C) Initial distribution of solvent-accessible surface area (SASA), hydrophilicity, and polarity features. (D to F) Distributions after model training. (G to I) Density plots of SASA, hydrophilicity, and polarity for both binding sites (red) and nonbinding sites (blue) with RSA > 0.05.

SpatConv shows a reduced reliance on certain physicochemical properties, which are typically important for predicting binding sites. Figure 6 and Fig. S14 clearly visualize various common physicochemical properties (SASA [43], hydrophilicity [44], polarity [45], isoelectric point [46], hydrophobicity [44], and charge [47]) of the protein before and after training. These properties are usually considered key features by previous predictors for distinguishing binding/nonbinding residues [42]. In contrast, our analysis suggests a limited role for these individual properties in SpatConv’s predictions. Specifically, Fig. 6A to C present the t-SNE visualizations of initial features colored by SASA, hydrophilicity, and polarity, respectively, while Fig. 6D to F show the feature distributions trained by SpatConv. As illustrated in Fig. 6, the pretrained features initially encode these physicochemical properties, but their influence diminishes during the training process. This indicates that SpatConv places relatively less emphasis on these properties while focusing more on learning the complex protein–protein binding interactions.

To further explore the relationship between physicochemical properties and binding site identification, we analyzed the distributions of these features for both binding and nonbinding residues. Given that binding sites are typically located on protein surfaces, we restricted our analysis to residues with RSA > 0.05. As shown in Fig. 6G to I, the density distributions of surface residues reveal similar patterns between binding and nonbinding sites across key physicochemical features. Comparable trends are observed for isoelectric point, hydrophobicity, and charge, as presented in Figs. S15 to S20. These results suggest that individual physicochemical properties alone may not strongly correlate with binding site identification in this context.

### Applying SpatConv to explore the SARS-CoV-2 spike protein

In this section, we predict the antibody binding sites of the SARS-CoV-2 spike glycoprotein using SpatConv. To predict the antibody binding sites, we used data from the Prefusion 2019-nCoV spike glycoprotein (PDB ID: 6VSB), which represents a conformation prior to receptor binding. We mapped the predicted binding residues of the unbound spike protein to the corresponding residues in the complex in order to clearly demonstrate the relationship between our predictions and the results of experimentally resolved antigen–antibody interactions (for a detailed description of the spike protein structure, see Note S3). This residue mapping was achieved by directly matching the residue IDs and chain identifiers between the unbound protein and the complex, as both structures are derived from the same sequence and maintain consistent residue numbering.

The spike protein is a key target for antibody-mediated neutralization, with the receptor binding domain (RBD) being the primary region interaction between the spike protein and the host cell ACE2 receptor [48]. Antibody binding to the RBD can obstruct its interaction with ACE2, preventing viral entry. The N-terminal domain (NTD), part of the spike protein’s S1 subunit [48,49], plays a key role in interactions with host cell receptors [50].

**Fig. 7.**
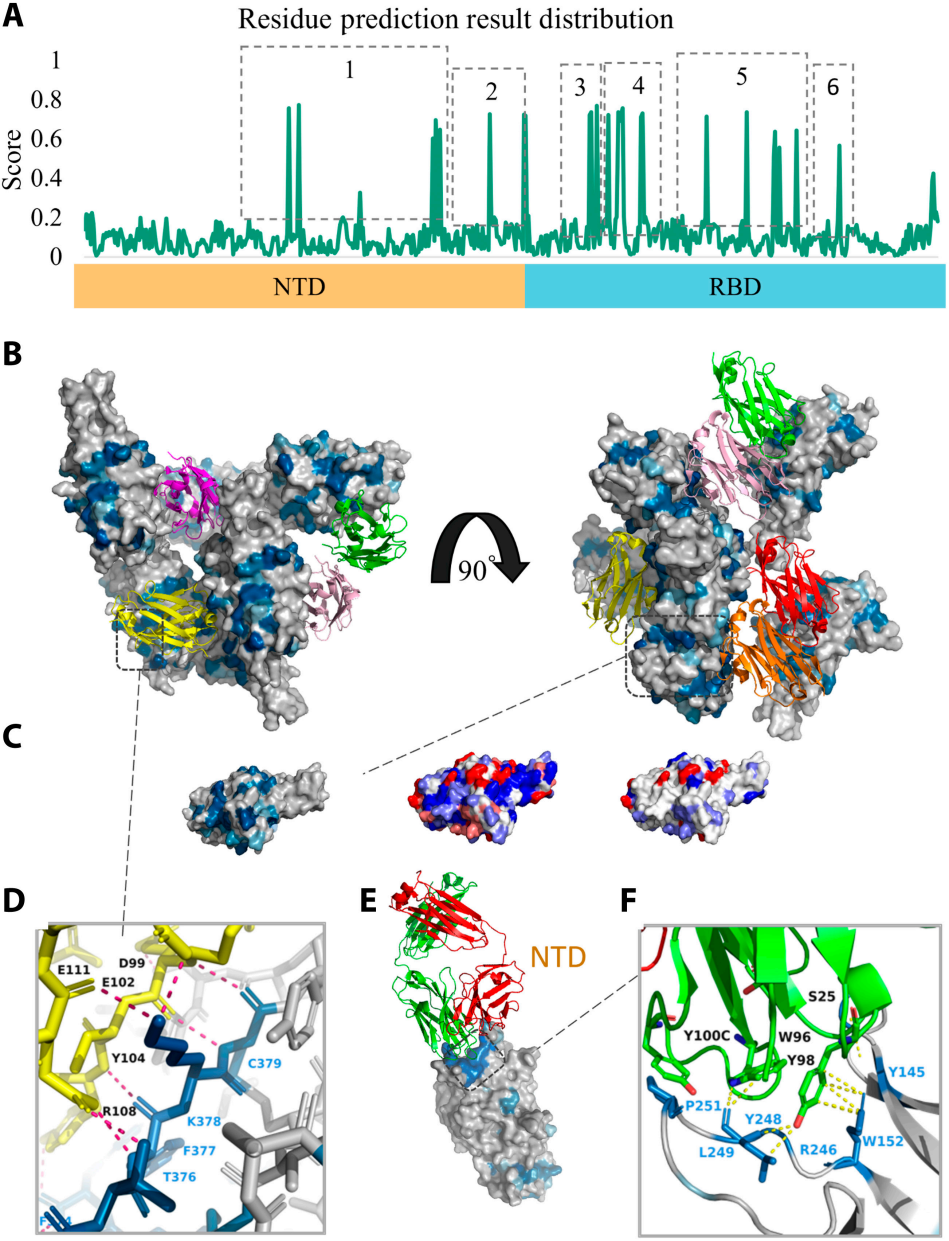
Visualization of binding sites on the severe acute respiratory syndrome coronavirus 2 (SARS-CoV-2) spike protein and prediction results. (A) Predicted scores on chain A of protein 6VSB. (B) Predicted scores for nanobody F2 binding to the receptor binding domain (RBD; Protein Data Bank [PDB] ID: 7OAY). (C) Predicted score (left), hydrophobicity values(middle), and electrostatic value (right) distribution in important regions. (D) Close-up view of the binding interface between the S1 RBD region of the spike protein and nanobody F2. (E) Predicted scores for neutralizing antibody 2-51 binding to the N-terminal domain (NTD; PDB ID: 7L2C). (F) Close-up view of the binding interface between 7L2C protein residues and neutralizing antibody 2-51. Key residues are shown as sticks. The protein surface is colored by predicted scores, ranging from low (gray) to high (deep blue). Antibodies binding to the main epitopes are displayed as colored cartoons.

Figure 7A shows the predicted binding sites for the RBD and NTD in chain A of the 6VSB protein, with 6 regions labeled. Regions 1, 3, 4, and 5 are well matched to experimentally verified or potentially binding sites with strong evidence [51,52], while regions 2 and 6 are predicted with high scores but lack sufficient evidence for confirmation.

For the RBD, SpatConv successfully predicts binding site residues from the unbound spike protein structure to their binding contact epitopes, particularly in relation to nanobody binding. Figure 7B shows the mapped binding residues on chains A, B, and C of the spike protein in the nanobody F2–RBD complex (PDB ID: 7OAY). The predicted binding region (region 4) closely overlaps with the experimentally resolved nanobody F2 binding region. Figure 7D provides a close-up of the binding interface, where all residues are correctly identified, including key residues (F377L and K378Q/N) involved in escape mutations [51].

Additionally, several potentially critical sites within the RBD have been identified [52]. For instance, residues such as Y489, F486, N487, and N501 have been reported as important for antibody binding and viral susceptibility (for more details on these sites and their roles, see Note S3). All of these critical sites were successfully identified within the predicted region 5. As illustrated in Fig. 7C (left), regions 3 and 5 constitute part of the RBD interface, which has a larger surface area than other antibody binding interfaces (such as H11-H4 RBD), enabling stronger hydrogen bonds, salt bridges and hydrophobic interactions [53]. Hydrophobicity (middle) and electrostatic potential (right) distributions on the surface of this region were visualized. Hydrophobicity analysis revealed clearly a hydrophobic nature, aiding interactions with the nanobody’s hydrophobic pocket, while the electrostatic potential map indicated regions with positive and negative charges, suggesting electrostatic interactions with the antibody. These results highlight the physicochemical properties that support the potential role of this region as a nanobody binding site in the RBD.

We also found that SpatConv predicts some residue sites in the NTD that may bind to neutralizing antibodies. The predicted mapping of neutralizing antibody 2-51 binding to the SARS-CoV-2 NTD (PDB ID: 7L2C) reveals a critical supersite (Fig. 7E), which includes residues Y145, W152, Y248, L249, and P251. A close-up of the predicted binding interface is shown in Fig. 7F. This supersite, located on the NTD periphery, features a dynamic β-hairpin and flexible loops, with a substantial positive charge, making it a known target for neutralizing antibodies [50] (for more details, see Note S3).

For the remaining predicted regions (2 and 6), there is insufficient evidence to confirm whether they are binding sites. Region 6, located within the RBD, may reveal additional binding information in future studies, while region 2, within the NTD, requires further experimental validation to assess its role in binding.

This study demonstrates the effectiveness of SpatConv in predicting antibody binding sites on the SARS-CoV-2 spike protein, offering new insights into protein–antibody interactions that could aid in antibody design and vaccine development.

## Discussion

This study shows that pretrained PLMs combined with the graph-free bio-spatial convolution techniques are highly effective in identifying protein binding sites at high resolutions. SpatConv outperforms many state-of-the-art graph-based or point cloud methods without needing explicit graph structure descriptions. It also avoids the computational overhead of precomputing or generating additional features, allowing for efficient analysis of large-scale protein structures. For instance, diffusion models can capture dynamic features in protein interaction networks [54], while large structural datasets from tools like AlphaFold 3 [55] can be analyzed quickly, enabling fast biological discoveries.

The contributions and innovations of SpatConv can be summarized as follows: (a) It introduces a graph-free bio-spatial convolution tailored for macromolecules, allowing the model to effectively capture residue representations and their spatial neighbors, providing a deeper understanding of protein structures and interactions. (b) Our model is specifically designed to jointly model the interactions between semantic features extracted from pretrained PLMs and local 3D spatial context, thereby enabling richer, more context-aware representations for accurate protein binding site prediction. (c) The innovative fusion of structural and sequence features markedly enhances the model’s ability to predict protein–ligand binding sites. By integrating the geometric context of residue positioning with the biochemical properties encoded in the sequence, this approach captures complex spatial dependencies that are essential for precise binding site identification. (d) SpatConv demonstrates robustness, showing near-identical performance on both ESMFold-predicted and experimental structures across diverse datasets (protein, peptide, Zn^2+^, and Mn^2+^), indicating its ability to tolerate protein conformational deviations. (e) Interpretability analysis explores the complex relationships between various physicochemical properties (e.g., charge and hydrophobicity) and protein binding sites. Contrary to commonly assumed correlations, we found that these properties do not strongly correlate with binding site identification in this context. This challenges previous assumptions and suggests that alternative features or more nuanced representations may be required for accurate binding site prediction. (f) SpatConv successfully predicts and visualizes antibody binding sites on the SARS-CoV-2 spike glycoprotein, identifying known regions on the RBD and NTD, as well as novel potential binding sites, thus providing valuable insights for antibody development and vaccine design.

In contrast to tools like AlphaFold that require both the protein and ligand information as input, SpatConv operates solely on the protein information, making it particularly suitable for scenarios where ligand data are unavailable, such as the study of newly discovered proteins or epitope identification. Moreover, AlphaFold-Multimer emphasizes complex-level structural accuracy, while SpatConv is tailored for precise binding site prediction, offering improved localization of functional regions. Additionally, SpatConv’s lightweight, graph-free architecture enables rapid and large-scale inference with very low computational cost, making it well-suited for high-throughput tasks. Together, these advantages position SpatConv as a complementary tool to AlphaFold, providing efficient and focused functional insights.

SpatConv features a versatile architecture that requires only a large corpus of training data to extend its applicability to domains such as protein–DNA and protein–RNA interactions. We believe that SpatConv will markedly advance the study of novel protein functions and mechanisms, offering strong technical support for drug design. Through an online server powered by SpatConv, we provide a user-friendly service for using the trained model at http://liulab.top/SpatConv/server. Users can predict protein binding sites by submitting query proteins (see Fig. S21 for the web server interface).

## Methods

### Preparation of benchmark datasets

Protein–protein binding site datasets were sourced from Protein–Protein Docking Benchmark 5.5 (Docking Benchmark Database [DBD] 5.5) [56] and Dockground [57], which provide high-resolution, nonredundant protein structures in both bound and unbound states. Since proteins often undergo conformational changes upon interaction with intracellular molecules, unbound structures were selected for model training and testing. An 80/20 split was used to allocate 80% of the data for training and 20% for testing.

To ensure data quality, sequence similarity clustering was performed using PSI-CD-HIT [58] on the redundant dataset, and the representative entry with the highest resolution (below 8.0 Å) was selected from each cluster. Following previous studies [33], a protein–protein binding residue was defined as a surface residue (RSA > 5%) whose SASA decreases by at least 1 Å^2^ upon binding. The protein binding site dataset comprised 1,001 proteins for the training set and 250 proteins for the test set. Additionally, we applied a temporal split to the protein–protein binding data using DBD version 5.5 as the cutoff for time segmentation. Proteins available in versions prior to 5.5 were used for training, while the 82 proteins introduced in version 5.5 and later were included in the test set. For proteins from Dockground, due to the absence of time information, they were randomly assigned to the training and test sets to maintain a 4:1 training-to-testing ratio.

Benchmark datasets for protein–ligand binding, including peptide binding and metal ion (Zn^2+^ and Mn^2+^) binding, were obtained from the BioLiP database [59], a curated collection of biologically relevant protein–ligand complexes primarily derived from the PDB. Protein chains were selected with resolutions ≤3.0 Å and lengths between 50 and 1,500 residues. A binding residue is defined if the smallest atomic distance between the target residue and the ligand is <0.5 Å plus the sum of the van der Waals radius of the 2 nearest atoms. Specifically for metal ion binding proteins, a residue was labeled as binding if the minimum atomic distance to the ligand was less than 0.5 Å + the sum of the van der Waals radii of the 2 nearest atoms.

To reduce redundancy, all datasets—protein–peptide and metal ion binding alike—were processed using CD-HIT to remove protein sequences with >25% sequence identity and >30% alignment coverage. After obtaining and processing all the 3 peptide and metal ion binding proteins, we used 2021 January 1 as the cutoff date for splitting the proteins into training and test sets. Proteins identified and deposited before this date were included in the training sets, while those discovered and recorded afterward were reserved for the test sets. Finally, the peptide binding site prediction dataset consisted of 1,251 protein chains in the training set and 235 chains in the test set. The Zn^2+^ and Mn^2+^ training sets contained 1,647 and 547 protein chains, respectively, with corresponding test sets of 211 and 57 chains. Detailed sample sizes and proportions of positive and negative samples are provided in Note S2.

### The SpatConv model

This section is organized as follows: First, we describe the extraction of sequence features for each residue. Next, we detail the extraction of structural features. We then explain the feature fusion mechanism of sequence and structural information. Finally, we discuss the implementation of bio-spatial convolution encoders for iterative residue representation learning. The bio-spatial convolution encoder is designed to extract residue-level features by jointly modeling sequence semantics and local 3D spatial geometry.

#### Sequence feature extraction

In our proposed end-to-end model, residue sequence features are extracted from the pretrained PLM ProtT5 (version: ProtT5-XL-U50) [21], a self-supervised autoencoder based on the transformer architecture, designed to extract protein sequence information. It was pretrained on the Big Fantastic Database dataset [60], which contains a large collection of protein sequences, allowing it to predict masked amino acids from sequence context. It was then fine-tuned on UniRef50, which includes over 500 million nonredundant sequences from various species and functions [61]. According to ProtT5, each amino acid receives an initial 1,024-dimensional feature vector $f^{0}$ . We then use the following formula to embed $f^{0}$ into a 64-dimensional vector f as the embedded sequence features of the residue:

$$f=\phi \left(W_{2}\left(\phi \left(W_{1}f^{0}+b_{1}\right)\right)+b_{2}\right)$$ (1)

where $\phi \left(\cdot \right)$ is the Leaky ReLU activation function, *W*_1_ and *W*_2_ are the learnable parameter matrices, and *b*_1_ and *b*_2_ are biases.

#### Structural feature extraction

To precisely represent structural characteristics, the coordinates of residues are extracted based on the 3D coordinates of their α carbons as recorded in the PDB file. SpatConv first builds a local sphere for each residue *i*, where those residues within the local sphere are defined as the spatial neighbors of *i* and these neighboring residues comprise the structural information of the local environment. In this study, the radius of the local sphere is set to 13 Å. A local coordinate system is then built for each local sphere to ensure SE(3) equivariance as follows: The coordinate origin is selected as the central residue *i*, and the *z* axis is defined by the α carbon and β carbon of residue *i* using the following formula:

$$z_{i}^{*}=\frac{C_{\beta_{i}}-C_{\alpha_{i}}}{\left\|C_{\beta_{i}}-C_{\alpha_{i}}\right\|}$$ (2)

where $C_{\alpha_{i}}$ and $C_{\beta_{i}}$ represent the coordinates of the α and β carbons of residue *i*, respectively, in the PDB file. Then, the *y* axis is defined using the *z* axis and the intermediate direction vector *d_i_* directing from the β carbon of residue *i* − 1 to the α carbon of residue *i* as follows:

$$y_{i}^{*}=\frac{z_{i}^{*}\times d_{i}}{\left\|z_{i}^{*}\times d_{i}\right\|}$$ (3)

$$d_{i}=\frac{C_{\beta_{i-1}}-C_{\alpha_{i}}}{\left\|C_{\beta_{i-1}}-C_{\alpha_{i}}\right\|}$$ (4)

where $C_{\beta_{i-1}}$ represents the coordinates of the β carbon of residue *i* − 1 in the original PDB file. For the first residue, in the absence of a preceding residue, a unit vector (1,0,0) in the coordinate system of the original PDB file is used as a substitute for the β carbon coordinate when calculating the *y* axis. Finally, the *x* axis is generated through the cross product of the *z* and *y* direction vectors as follows:

$$x_{i}^{*}=\frac{z_{i}^{*}\times y_{i}^{*}}{\left\|z_{i}^{*}\times y_{i}^{*}\right\|}$$ (5)

After the construction of the local coordinate system, each neighboring residue j in the local sphere is assigned local coordinates $\left(x_{j},\;y_{j},\;z_{j}\right)$, and the constructed local coordinate systems satisfy rotational and translational invariance.

$$\left(x_{j},\;y_{j},\;z_{j}\right)=P_{j}^{*}\cdot \left(x_{i}^{*},\;y_{i}^{*},\;z_{i}^{*}\right)$$ (6)

$$P_{j}^{*}=C_{\alpha_{j}}-C_{\alpha_{i}}$$ (7)

where $P_{j}^{*}$ is the relative position vector between residue i and residue j defined by the coordinates of the 2 residues in the PDB file.

#### Feature fusion and bio-spatial convolution

In protein binding site prediction, the spatial position and orientation information of residues, as well as their sequence features, are critical for understanding protein functions and interactions. To effectively capture and integrate these pieces of information, we propose a bio-spatial convolutional encoder that integrates local spatial geometry with residue-level semantic features.

This encoder processes residues in 3 stages: spatially guided modulation, feature aggregation with Gaussian weighting, and residual-enhanced updates.

1. Spatially guided feature modulation. Each residue j is first represented by its 3D local coordinates $\left(x_{j},\;y_{j},\;z_{j}\right)\in R^{n\times 3}$ and its embedded sequence feature $f_{j}\in R^{n\times 64}$. To incorporate spatial awareness into the semantic space, we perform element-wise multiplication between each coordinate dimension and the corresponding sequence embedding:

$$f_{j}^{\left(x\right)}=x_{j}⊙f_{j},\;f_{j}^{\left(y\right)}=y_{j}⊙f_{j},\;f_{j}^{\left(z\right)}=z_{j}⊙f_{j}$$ (8)

This operation allows each spatial channel to act as a directional weight over the semantic features, enhancing the model’s ability to learn geometry-aware representations.

The resulting vectors are concatenated across all 3 channels to form an enriched spatial–semantic representation:

$$f_{j}^{\text{concat}}=\left[f_{j}^{\left(x\right)};\;f_{j}^{\left(y\right)};\;f_{j}^{\left(z\right)}\right]$$ (9)

This fused representation is then passed through an MLP with nonlinear activation to capture higher-order interactions:

$${\hat{f}}_{j}=\mathrm{MLP}\left(f_{j}^{\text{concat}}\right)$$ (10)

Here, ${\hat{f}}_{j}\in {\mathbb{R}}^{n\times 64}$ denotes the updated representation of residue j, preserving both semantic and directional spatial information.

By applying element-wise multiplication between the residue’s 3D coordinates and its sequence embedding, we directly modulate each embedding dimension with spatial location information. This approach enables the model to learn how spatial positioning regulates semantic features. It preserves the original semantic richness of the sequence embedding while embedding geometric awareness into the representation, thereby improving the model’s ability to capture residue binding patterns.

2. Gaussian-weighted feature aggregation. To control the influence of neighboring residues in the spatial domain, we adopt a Gaussian weighting function, which is widely used for smoothing and distance-based weighting. This function assigns larger weights to closer residues and progressively smaller weights to those farther away, aligning well with biological intuition that spatial proximity correlates with interaction likelihood.

$$G\left(d_{j}\right)=\exp\left(-\frac{d_{j}^{2}}{2\sigma^{2}}\right)$$ (11)

where *d_j_* is the distance between the central residue *i* and its neighbor *j* and *σ* is a scaling parameter. Regarding the choice of *σ*, we set its default value to align with the residue neighborhood threshold, thereby balancing the contributions of nearby and distant residues. Furthermore, we conducted a parameter sweep across several candidates (e.g., *σ* = 12, 13, 14, and 15 Å) and evaluated the model’s performance under each setting. Results showed that SpatConv is not sensitive to these values and demonstrates similar performance with different values of *σ* (see Tables S6 to S9 and Figs. S4 to S7). The aggregated feature ${\hat{f}}_{i}^{l}$ of residue *i* at layer *l* is computed by a weighted sum of its neighbors’ features, followed by batch normalization:

$${\hat{f}}_{i}^{l}=\mathrm{BN}\left(\sum_{j=1}^{N}G\left(d_{j}\right){\hat{f}}_{j}^{l-1}\right)$$ (12)

where 𝑁 is the number of neighboring residues.

3. Residual feature update and identity mapping. To preserve the initial semantic context while allowing for depth-wise refinement, we apply a residual connection between the current aggregated feature and the original input embedding $f_{i}^{0}$:

$$h_{i}^{l}=\left(1-\alpha \right){\hat{f}}_{i}^{l}+\text{αMLP}\left(f_{i}^{0}\right)$$ (13)

A gated identity mapping mechanism is then used to balance transformation and identity preservation:

$$f_{i}^{l}=\varphi \left(h_{i}^{l}\left(\theta_{l}W+\left(1-\theta_{l}\right)I_{n}\right)+{\hat{f}}_{i}^{l}\right)$$ (14)

Here, φ denotes the ReLU activation, *W* is a learnable weight matrix, and $I_{n}$ is the identity matrix. The gating coefficient $\theta_{l}$ varies with layer depth *l*, defined as

$$\theta_{l}=\text{min}\left(1,\;\text{log}\left(\frac{\lambda }{l}+1\right)\right)$$ (15)

Hyperparameters are set to *α* = 0.7 and *λ* = 1.5, ensuring a smooth transition from identity preservation to transformation over layers.

#### Residue classification and optimization

After 3 layers of bio-spatial convolutions, the final feature representation $f_{i}^{l}$ for each residue is passed through a 2-layer MLP classifier with ReLU and sigmoid activations to produce a binding probability:

$$\hat{y}=\sigma \left(W_{2}\left(\phi \left(W_{1}f_{i}^{l}+b_{1}\right)\right)+b_{2}\right)$$ (16)

The model is trained using binary cross-entropy loss, optimized via the Adam optimizer. We set the learning rate to 10^−3^, batch size to 1, and maximum epochs to 20. Learning rate scheduling and early stopping are employed based on validation performance. For implementation details, including environment setup and runtime statistics, please refer to Note S4.

## Data Availability

All data resources used are freely available from the following databases: DBD 5.5 (https://zlab.umassmed.edu/benchmark) and Dockground (http://dockground.compbio.ku.edu/). The processed data used for training and testing the models in this study can be available at https://zenodo.org/records/10826801. The source code files for reproducing and evaluating SpatConv is available at the GitHub repository https://github.com/gmnnnhh/SpatConv.

## Supplementary Materials

Supplementary 1 Notes S1 to S5 Tables S1 to S10 Figs. S1 to S22
