# Supplementary material for: SpatConv Enables the Accurate Prediction of Protein Binding Sites by a Pretrained Protein Language Model and an Interpretable Bio-spatial Convolution
Source: Research (Wash D C). 2025 Jul 8;8:0773. doi: 10.34133/research.0773 (PMC12237623; doi:10.34133/research.0773)
Supplement: Supplementary 1 — Notes S1 to S5 Tables S1 to S10 Figs. S1 to S22 [file research.0773.f1.pdf]

Supplementary Information

SpatConv Enables the Accurate Prediction of Protein Binding Sites by a Pretrained Protein Language Model and an Interpretable Bio-Spatial Convolution

Mingming Guan[1,†], Jiyun Han[1,†], Shizhuo Zhang[1], Hongyu Zheng[2,*], Juntao Liu[1,*]

[1]School of Mathematics and Statistics, Shandong University (Weihai), Weihai, 264209, China

[2]Department of Radiation Oncology, Qilu Hospital, Cheeloo College of Medicine, Shandong University, Jinan, 250012, China

[†]These authors contributed equally to this study
[*]Address correspondence to: Hongyu Zheng; Zhenghy1308@163.com and Juntao Liu; juntaosdu@126.com

Table of Content

Supplementary Notes

Supplementary Tables

Supplementary Figures

Supplementary Notes

Supplementary Note 1 | Performance Metrics.

In this study we used the following evaluation metrics to measure the predicted performance: Precision, Recall, F1-score (F1), Matthews correlation coefficient (MCC), area under the receiver operating characteristic curve (AUROC), area under the precision-recall curve (AUPRC), and accuracy (ACC).

$$Precision = \frac{TP}{TP + FP}$$

$$Recall = \frac{TP}{TP + FN}$$

$$F1 = 2 \times \frac{Precision \times Recall}{Precision + Recall}$$

$$MCC = \frac{TP \times TN - FN \times FP}{\sqrt{(TP + FP) \times (TP + FN) \times (TN + FP) \times (TN + FN)}}$$

$$ACC = \frac{TP + TN}{TP + FP + FN + TN}$$

where true positives (TP) and true negatives (TN) denote the number of interaction and noninteraction sites identified correctly, and false positives (FP) and false negatives (FN) denote the number of falsely predicted interaction and noninteraction sites, respectively. The AUROC is calculated as the area under the ROC curve. A ROC curve shows the trade-off between true positive rate (TPR) and false positive rate (FPR) across different decision thresholds. The AUPRC is calculated as the area under the PR curve. A PR curve is a graph with Precision values on the y-axis and Recall values on the x-axis across different decision thresholds.

Supplementary Note 2 | Dataset Overview: Protein-Protein, Peptide, Mn^{2+}, and Zn^{2+} Binding Sites

The protein-protein binding sites dataset contains 289,736 residues in total, including 25,172 positive samples (i.e., binding sites based on the definition of interacting residues) and 264,564 negative samples. The peptide binding sites dataset is divided into a training set and a test set. The training set consists of 1,251 sequences with 348,370 residues, 5.39% of which are binding residues. The test set includes 235 sequences with 74,788 residues, where 4.50% are binding residues. The Mn^{2+} binding sites dataset is also split into a training set and a test set. The training set comprises 547 sequences with 181,699 residues, 1.41% of which are binding residues. The test set contains 57 sequences with 20,419 residues, where 1.10% are binding residues. The Zn^{2+} binding sites dataset includes a training set of 1,646 sequences with 474,855 residues, of which 1.63% are binding residues, and a test set of 211 sequences with 56,020 residues, where 1.85% are binding residues.

Supplementary Note 3 | Structural and Functional Overview of the SARS-CoV-2 Spike

The spike protein (S) of SARS-CoV-2, a homotrimer, plays a crucial role in viral entry by mediating interactions with host cell receptors, particularly ACE2. Each of the three polypeptide chains that form the spike protein contributes to its structure[1], with one receptor-binding domain (RBD) often adopting an "up" conformation to facilitate receptor binding. Several key sites within the spike protein have been implicated in viral infectivity and antibody neutralization. The RBD (PDB ID: 7OAY) contains critical residues, including Y489, F486, N487, and N501, which influence the virus's interaction with neutralizing antibodies and host receptors.

The N-terminal domain (NTD) of the spike protein, part of the S1 subunit, is another major target for neutralizing antibodies[2,3]. The NTD contributes to interactions with host cell surface receptors and exhibits unique electrostatic and conformational features. Structural analysis predicts a key supersite within the NTD (PDB ID: 7L2C) composed of residues Y145, W152, Y248, L249, and P251. This supersite, characterized by a dynamic β-hairpin structure and flexible loops, is positioned on the periphery of the NTD, away from the viral membrane and free from glycan modifications. Its high positive charge and structural dynamics make it a prime target for neutralizing antibodies, which are critical in preventing viral infection[4].

Understanding the structure and function of these critical sites in the spike protein, particularly the RBD and NTD, is vital for developing targeted therapeutics and vaccines aimed at neutralizing SARS-CoV-2. The identification of potent binding sites and their role in viral infectivity can aid in the design of future antiviral strategies.

Supplementary Note 4 | Environment Configurations and Running Time Tests

In terms of software and runtime, the model was implemented in Python using the following scientific computing and machine learning packages: Python[5] v.3.8.13; numpy v.1.24.4; h5py[6] v.3.10.0; PyTorch[7] v.1.12; Biopython[8] v.1.83; numba[9] v.0.58.1; pandas v.2.0.3; scipy[10] v.1.10.1; matplotlib v.3.7.5 and scikit-learn[11] v.1.3.2.

All experiments were conducted on the following server configuration:

GPU: NVIDIA RTX A6000 (Driver Version: 525.105.17, CUDA Version: 12.0, 48 GB VRAM);

CPU: Dual Intel(R) Xeon(R) Silver 4210R CPUs @ 2.40GHz, 40 threads total; RAM: 65.5 GB.

Supplementary Note 5 | Inference and Computation Time Comparisons Across Methods

We benchmarked the average inference time per protein for several representative methods, excluding costly preprocessing steps where applicable. SpatConv achieves the fastest inference at 0.016 seconds, excluding ProtT5 embedding generation (which itself takes 0.06 seconds per protein). In comparison, Spatom requires 0.04 seconds (excluding PSSM and DSSP), and GraphRRIS takes 0.362 seconds (excluding PSSM, HMM, and DSSP). ScanNet

is notably slower at 27.27 seconds, excluding MSA computation, while GraphBind takes 9.155 seconds under similar exclusions. MaSIF is the slowest, averaging 120.68 seconds, including surface calculations. For reference, the average preprocessing time per protein is 319.04 seconds for MSA, 754.06 seconds for PSSM, 101.63 seconds for HMM, and 0.028 seconds for DSSP.

Supplementary Tables

Table S1. Performance of SpatConv and other compared methods on Test set.

Test set	Method	Recall	Precision	F1-score	MCC	AUROC	AUPRC
Protein	GraphPPIS	0.429	0.228	0.298	0.230	0.751	0.212
	MaSIF-site	**0.515**	0.170	0.255	0.184	0.732	0.179
	ScanNet	0.415	0.285	0.338	0.275	0.797	0.258
	PeSTo	0.373	0.327	0.349	0.278	0.788	0.273
	Spatom	0.480	0.299	0.369	0.310	0.816	0.336
	SpatConv*	0.428	**0.378**	0.402	0.347	0.825	0.361
	SpatConv	0.487	0.370	**0.421**	**0.364**	**0.835**	**0.386**
Peptide	PepNN-Seq	0.289	0.153	0.200	0.158	0.729	0.128
	PepBind	0.062	**0.576**	0.112	0.178	0.655	0.148
	PepNN	0.351	0.180	0.238	0.202	0.765	0.163
	PepBCL	0.168	0.389	0.234	0.233	0.758	0.222
	SpatConv*	0.295	0.263	0.290	0.254	0.767	0.235
	SpatConv	**0.308**	**0.284**	**0.295**	**0.261**	**0.785**	**0.241**
Zn^{2+}	MIB	0.744	0.219	0.339	0.385	0.935	0.394
	TargetS	0.454	0.749	0.566	0.578	0.874	0.593
	lonCom*	0.849	0.145	0.248	0.327	0.939	0.676
	lonCom	0.852	0.137	0.236	0.317	0.937	0.671
	SpatConv*	**0.709**	0.775	0.741	0.737	0.971	0.775
	SpatConv	0.676	**0.849**	**0.753**	**0.754**	**0.972**	**0.783**
Mn^{2+}	MIB	0.462	0.096	0.159	0.193	0.856	0.168
	lonCom	0.511	0.245	0.331	0.344	0.833	0.304
	TargetS	0.271	0.496	0.351	0.362	0.864	0.322
	GraphBind	0.378	**0.644**	0.476	0.489	0.928	0.473
	SpatConv*	0.582	0.587	0.585	0.580	0.933	0.523
	SpatConv	**0.582**	0.615	**0.600**	**0.594**	**0.953**	**0.545**

Note: Methods marked with * correspond to evaluations performed using the ESMFold-predicted structures as input.

Table S2. Performance Metrics of Ablation Studies with Different Settings on Protein.

**Ablation**	Recall	Precision	F1-score	MCC	AUROC	AUPRC
Remove residues (RSA<0.05)	0.460	0.355	0.401	0.341	0.806	0.363

Remove local coordinate systems	0.409	0.352	0.378	0.318	0.813	0.347
Remove Gaussian distances	0.473	0.356	0.406	0.347	0.824	0.374
Remove all structural information	0.423	0.337	0.375	0.313	0.811	0.344
Use Hadamard product	0.421	0.365	0.391	0.331	0.820	0.363
Use ProstT5 embeddings	0.492	0.330	0.395	0.339	0.823	0.360
SpatConv	**0.487**	**0.370**	**0.421**	**0.364**	**0.835**	**0.386**

Table S3. Performance Metrics of Ablation Studies with Different Settings on Peptides.

**Ablation**	Recall	Precision	F1-score	MCC	AUROC	AUPRC
Remove residues (RSA<0.05)	0.291	0.259	0.274	0.238	0.782	0.211
Remove local coordinate systems	0.287	0.264	0.264	0.227	0.763	0.204
Remove Gaussian distances	**0.324**	0.238	0.274	0.238	0.771	0.196
Remove all structural information	0.299	0.226	0.258	0.220	0.762	0.198
Use Hadamard product	0.288	0.250	0.268	0.231	0.763	0.204
Use ProstT5 embeddings	0.259	0.232	0.245	0.208	0.754	0.180
SpatConv	0.308	**0.284**	**0.295**	**0.261**	**0.785**	**0.241**

Table S4. Performance Metrics of Ablation Studies with Different Settings on Zn^{2+} Ion.

**Ablation**	Recall	Precision	F1-score	MCC	AUROC	AUPRC
Remove residues (RSA<0.05)	0.709	0.775	0.740	0.739	0.968	0.766
Remove local coordinate systems	0.644	0.790	0.710	0.708	0.965	0.728
Remove Gaussian distances	**0.680**	0.804	0.743	0.741	**0.973**	0.781
Remove all structural information	0.612	0.824	0.704	0.703	0.967	0.735
Use Hadamard product	0.638	0.803	0.711	0.711	0.967	0.743
Use ProstT5 embeddings	0.607	0.809	0.694	0.696	0.930	0.686
SpatConv	0.676	**0.849**	**0.753**	**0.754**	0.972	**0.783**

Table S5 Performance Metrics of Ablation Studies with Different Settings on Mn^{2+} Ion.

**Ablation**	Recall	Precision	F1-score	MCC	AUROC	AUPRC
Remove residues (RSA<0.05)	0.582	0.577	0.576	0.571	0.926	0.528
Remove local coordinate systems	0.520	0.606	0.560	0.557	0.951	0.497
Remove Gaussian distances	0.587	0.569	0.589	0.587	0.951	0.511
Remove all structural information	0.484	**0.649**	0.555	0.556	0.951	0.507
Use Hadamard product	0.582	0.541	0.561	0.556	**0.960**	0.491
Use ProstT5 embeddings	0.498	0.552	0.523	0.519	0.937	0.447
SpatConv	**0.582**	0.615	**0.600**	**0.594**	0.953	**0.545**

Table S6. Performance Metrics of Ablation Studies under Different Parameter Settings on Protein.

**Ablation**		Recall	Precision	F1-score	MCC	AUROC	AUPRC
Structural neighborhood radius	12	0.432	0.390	0.409	0.356	0.831	0.373
	**13**	**0.487**	**0.370**	**0.421**	**0.364**	**0.835**	**0.386**
	14	0.444	0.386	0.413	0.356	0.828	0.382
	15	0.473	0.359	0.408	0.350	0.828	0.381
Dropout rates	**0.2**	**0.487**	**0.370**	**0.421**	**0.364**	**0.835**	**0.386**
	0.3	0.488	0.352	0.409	0.351	0.830	0.367
	0.4	0.454	0.371	0.408	0.350	0.823	0.378
	0.5	0.493	0.366	0.420	0. 363	0.836	0.387
Number of representation layers	1	0.466	0.375	0.415	0.358	0.828	0.385
	2	0.474	0.367	0.413	0.356	0.825	0.380
	**3**	**0.487**	**0.370**	**0.421**	**0.364**	**0.835**	**0.386**
Dimensionality of the embedding feature vector	**64**	**0.487**	**0.370**	**0.421**	**0.364**	**0.835**	**0.386**
	128	0.474	0.351	0.403	0.344	0.824	0.376
	256	0.426	0.369	0.395	0.337	0.816	0.361

Table S7. Performance Metrics of Ablation Studies under Different Parameter Settings on Peptides.

**Ablation**		Recall	Precision	F1-score	MCC	AUROC	AUPRC
Structural neighborhood radius	12	0.289	0.255	0.270	0.234	0.778	0.213
	13	0.295	0.284	0.289	0.255	0.767	0.233
	14	**0.325**	0.248	0.281	0.245	0.774	0.219
	**15**	0.308	**0.284**	**0.295**	**0.261**	**0.785**	**0.241**
Dropout rates	0.2	0.294	0.270	0.282	0.247	0.770	0.224
	**0.3**	**0.308**	**0.284**	**0.295**	**0.261**	**0.785**	**0.241**
	0.4	0.307	0.263	0.283	0.248	0.765	0.226
	0.5	0.294	0.270	0.282	0.247	0.770	0.224
Number of representation layers	1	**0.324**	0.238	0.274	0.238	0.771	0.196
	2	0.280	**0.287**	0.284	0.250	0.784	0.233
	**3**	0.308	0.284	**0.295**	**0.261**	**0.785**	**0.241**
Dimensionality of the embedding feature vector	64	0.307	0.263	0.283	0.248	0.765	0.226
	**128**	0.308	**0.284**	**0.295**	**0.261**	**0.785**	**0.241**
	256	**0.350**	0.231	0.279	0.243	0.777	0.227

Table S8. Performance Metrics of Ablation Studies under Different Parameter Settings on Zn^{2+} Ion.

**Ablation**		Recall	Precision	F1-score	MCC	AUROC	AUPRC
Structural neighborhood radius	12	0.674	0.790	0.727	0.725	0.969	0.762
	**13**	0.676	**0.849**	**0.753**	**0.754**	0.972	0.783
	14	**0.679**	0.819	0.742	0.741	**0.974**	**0.784**
	15	0.671	0.839	0.746	0.746	0.971	0.776
Dropout rates	0.2	**0.679**	0.804	0.737	0.735	0.968	0.763
	0.3	0.672	0.849	0.750	0.751	0.971	0.778
	0.4	0.662	0.834	0.738	0.739	0.968	0.767
	**0.5**	0.676	**0.849**	**0.753**	**0.754**	**0.972**	**0.783**
Number of representation layers	**1**	**0.676**	**0.849**	**0.753**	**0.754**	**0.972**	**0.783**
	2	0.662	0.834	0.738	0.739	0.968	0.767
	3	0.659	0.842	0.739	0.741	0.971	0.770
Dimensionality of the embedding feature vector	**64**	0.676	**0.849**	**0.753**	**0.754**	**0.972**	**0.783**
	128	**0.680**	0.798	0.735	0.732	0.971	0.763
	256	0.671	0.848	0.749	0.750	0.971	0.775

Table S9. Performance Metrics of Ablation Studies under Different Parameter Settings on Mn^{2+} Ion.

**Ablation**		Recall	Precision	F1-score	MCC	AUROC	AUPRC
Structural neighborhood radius	12	0.542	0.581	0.561	0.556	0.954	0.521
	13	**0.609**	0.537	0.571	0.567	0.951	0.502
	**14**	0.582	**0.615**	**0.600**	**0.594**	**0.953**	**0.545**
	15	0.582	0.541	0.561	0.556	0.960	0.491
Dropout rates	0.2	0.516	**0.630**	0.567	0.566	0.938	0.510
	0.3	0.582	0.577	0.580	0.575	0.952	0.497
	0.4	**0.609**	0.537	0.571	0.567	0.951	0.502
	**0.5**	0.582	0.615	**0.600**	**0.594**	**0.953**	**0.545**
Number of representation layers	**1**	0.582	**0.615**	**0.600**	**0.594**	**0.953**	**0.545**
	2	0.582	0.577	0.580	0.575	0.952	0.517
	3	**0.604**	0.600	0.598	0.594	0.952	0.525
Dimensionality of the embedding feature vector	**64**	0.582	**0.615**	**0.600**	**0.594**	**0.953**	**0.545**
	128	0.560	0.581	0.570	0.566	0.938	0.465
	256	**0.600**	0.508	0.550	0.546	0.941	0.488

Table S10. Performance of SpatConv and other compared methods on new Test set.

**Test set**	**Method**	**Recall**	**Precision**	**F1-score**	**MCC**	**AUROC**	**AUPRC**
Protein	GraphPPIS	0.435	0.221	0.293	0.227	0.748	0.196
	MaSIF-site	**0.504**	0.156	0.238	0.163	0.711	0.167
	ScanNet	0.428	0.297	0.350	0.29	0.803	0.265
	PeSTo	0.358	0.324	0.340	0.275	0.777	0.256
	Spatom	0.384	0.297	0.335	0.262	0.750	0.284
	SpatConv	0.447	**0.423**	**0.435**	**0.385**	**0.838**	**0.409**

Supplementary Figures

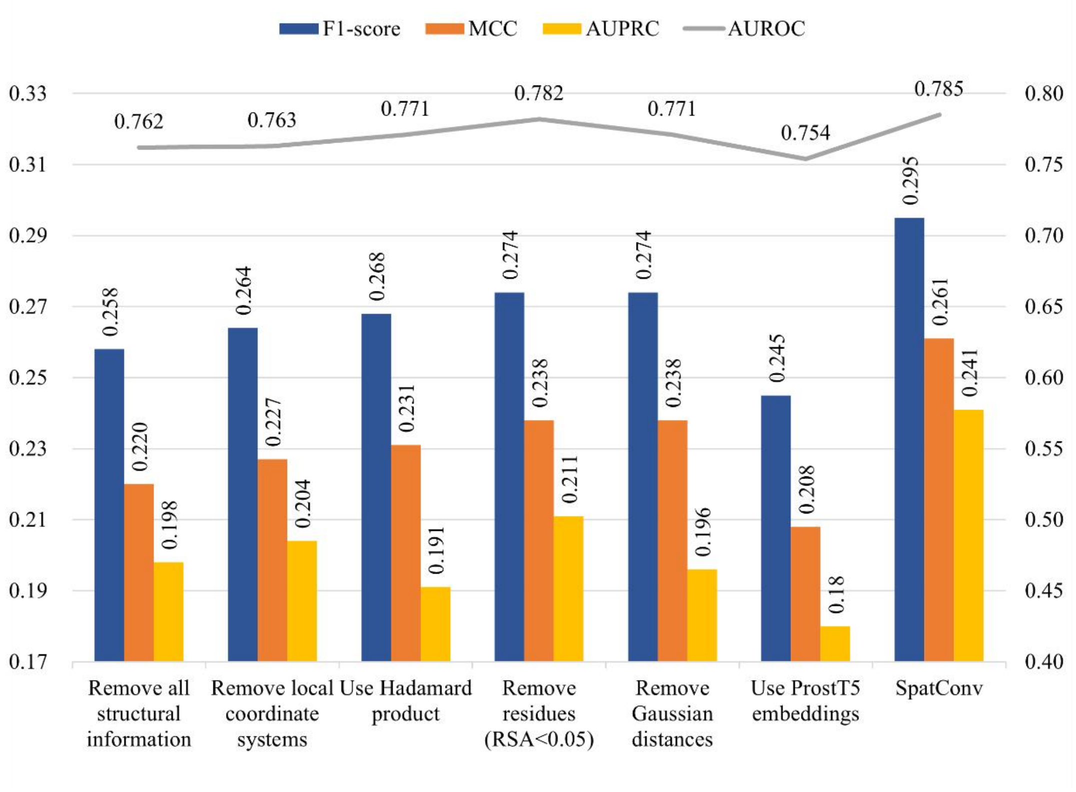

Figure S1 Performance Comparison of Different Ablation Methods for Peptide.

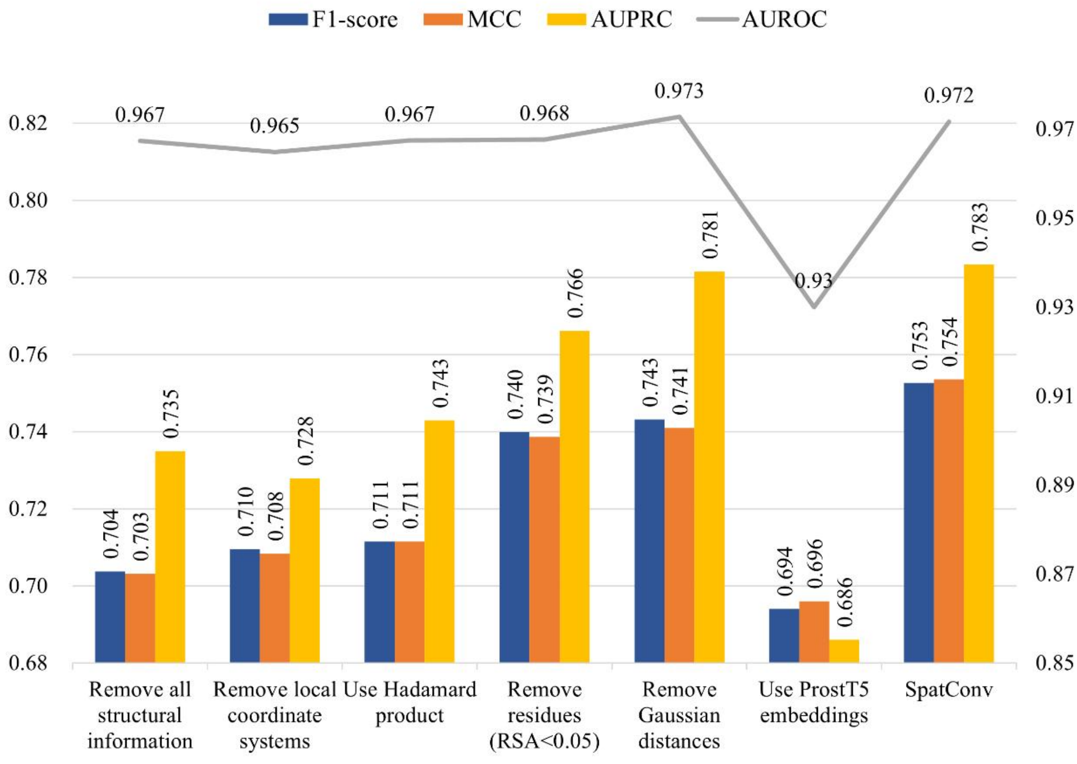

Figure S2 Performance Comparison of Different Ablation Methods for Zn^{2+} Ion.

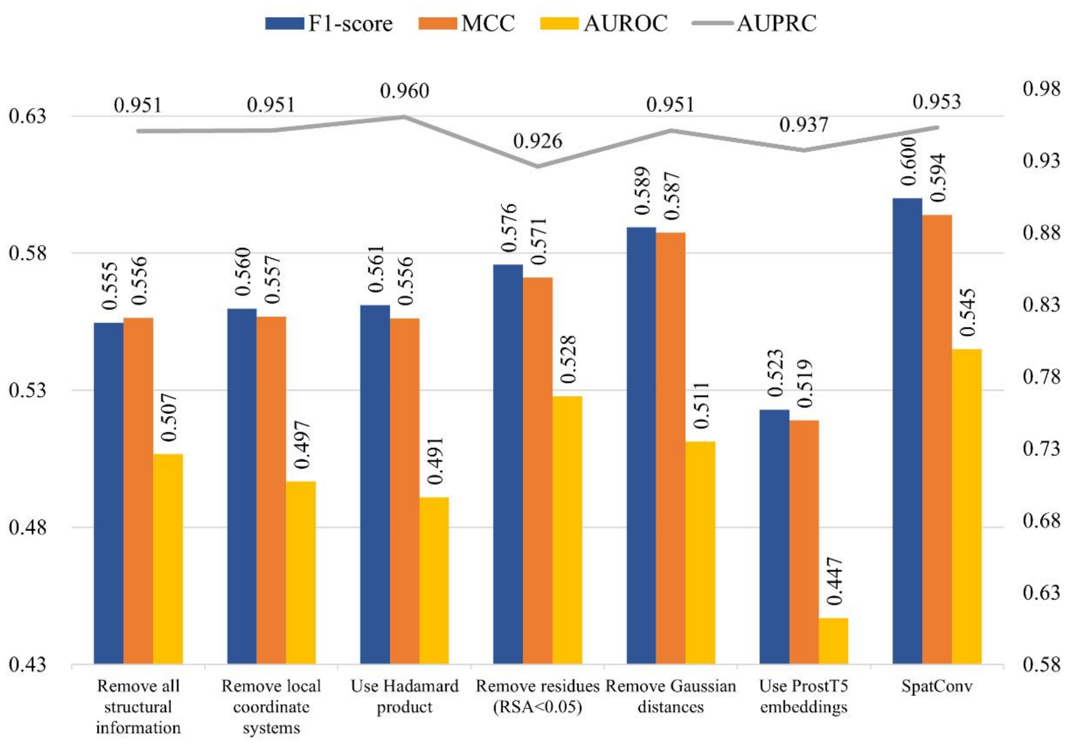

Figure S3 Performance Comparison of Different Ablation Methods for Mn^{2+} Ion.

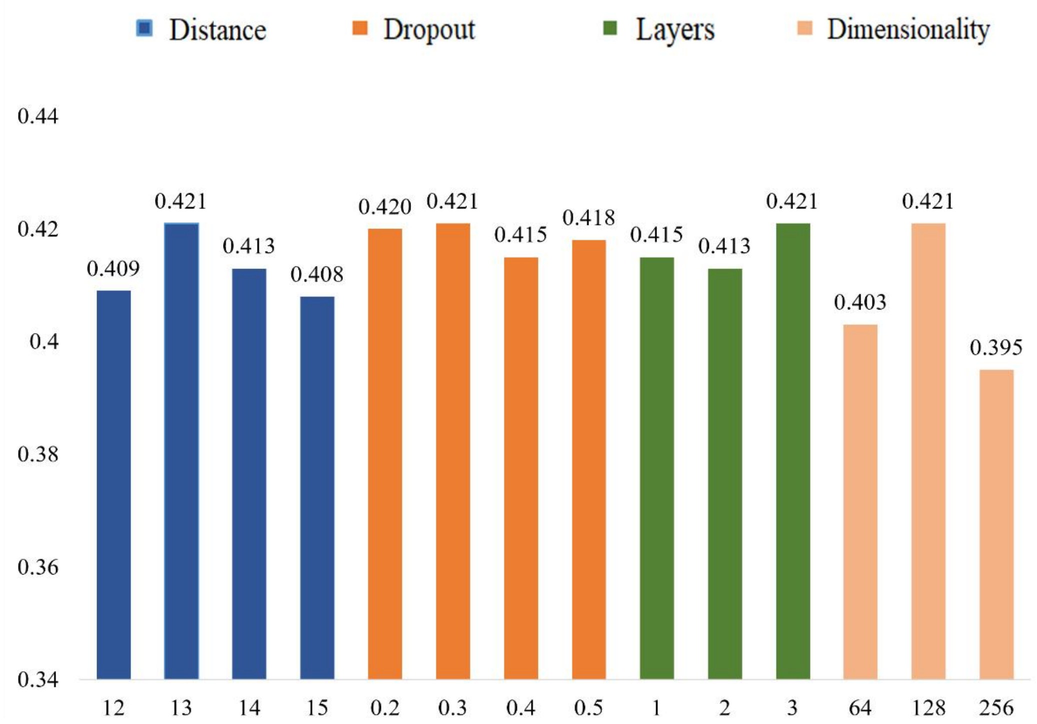

Figure S4 Trends of F1, MCC, and PRC Scores for protein under Different Parameters.

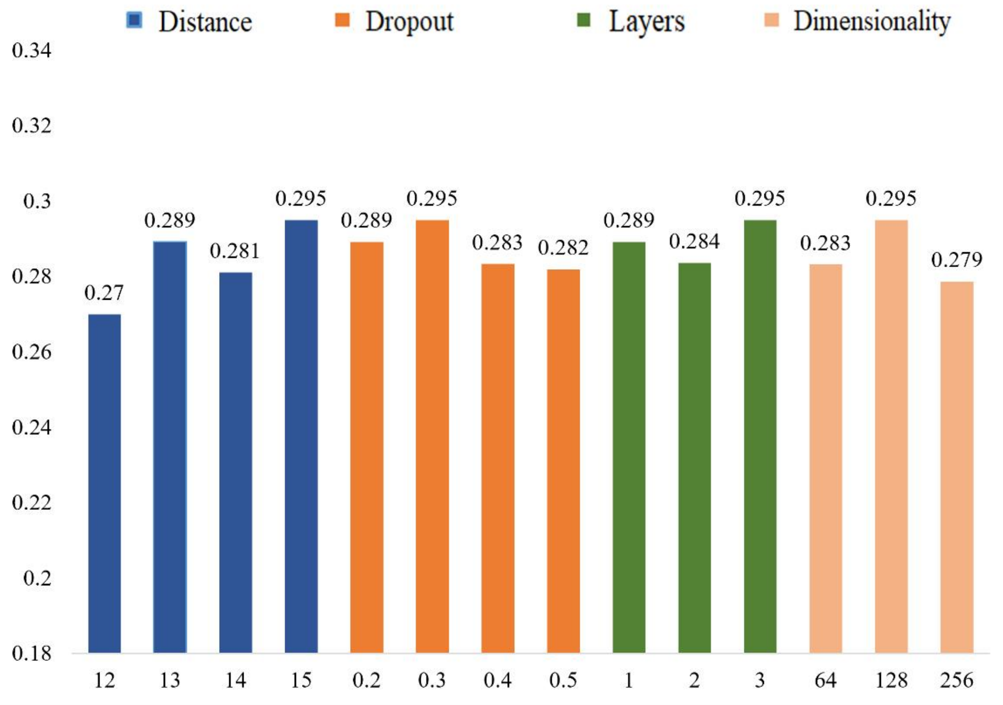

Figure S5 Trends of F1, MCC, and PRC Scores for peptide under Different Parameters.

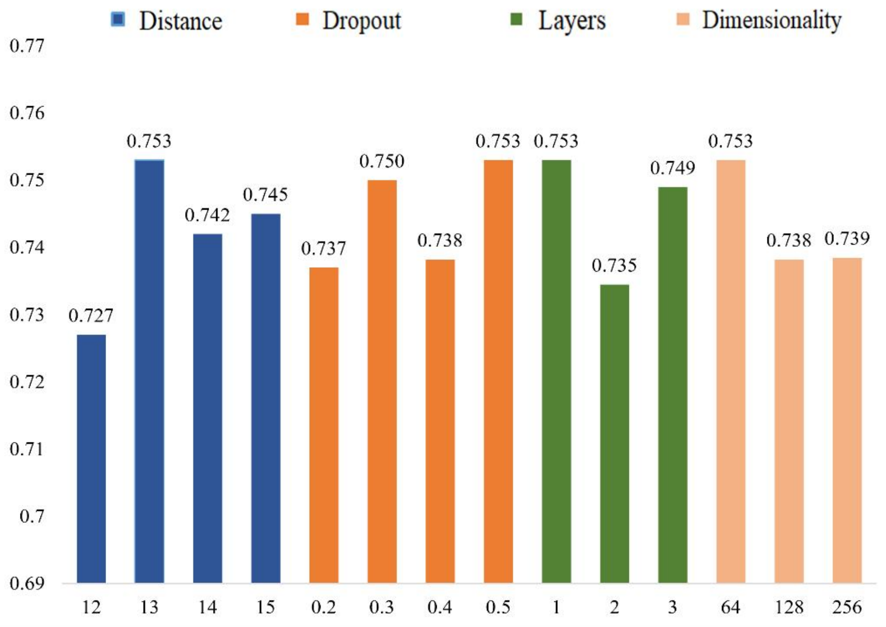

Figure S6 Trends of F1, MCC, and PRC Scores for Zn^{2+} Ion under Different Parameters.

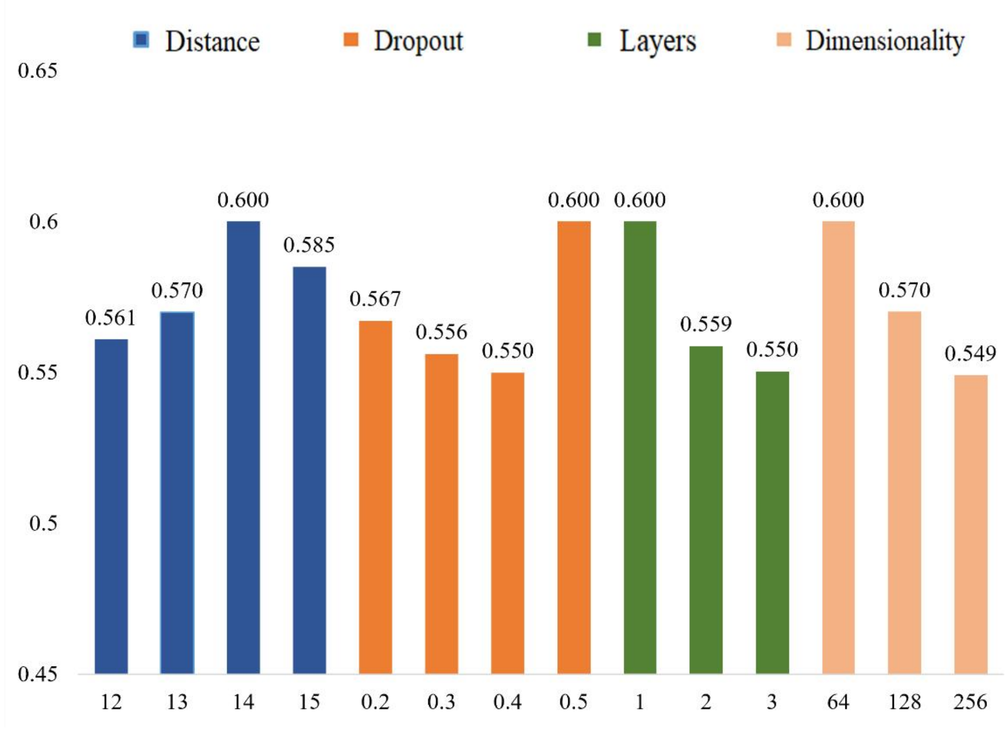

Figure S7 Trends of F1, MCC, and PRC Scores for Mn^{2+} Ion under Different Parameters.

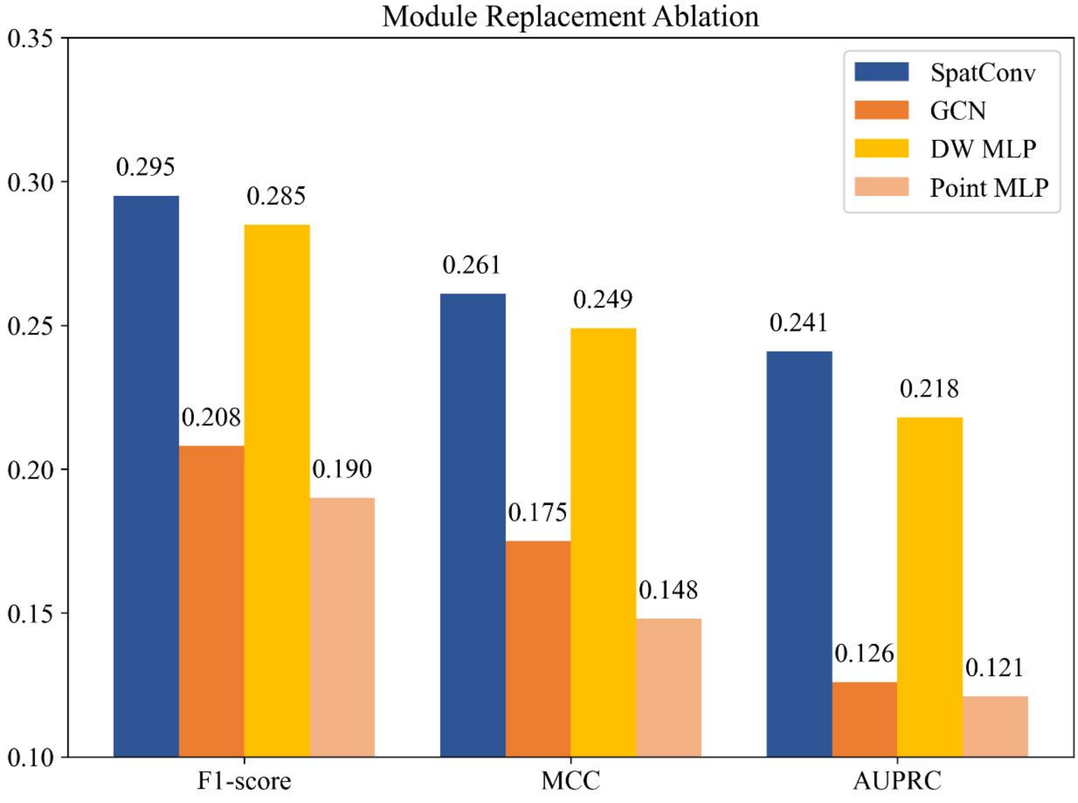

Figure S8 Module replacement ablation study of the feature aggregation component: The proposed SpatConv is compared against three alternatives—GCN (graph-based), DW MLP (distance-aware but graph-free), and Point MLP (fully graph-free)—for peptide data.

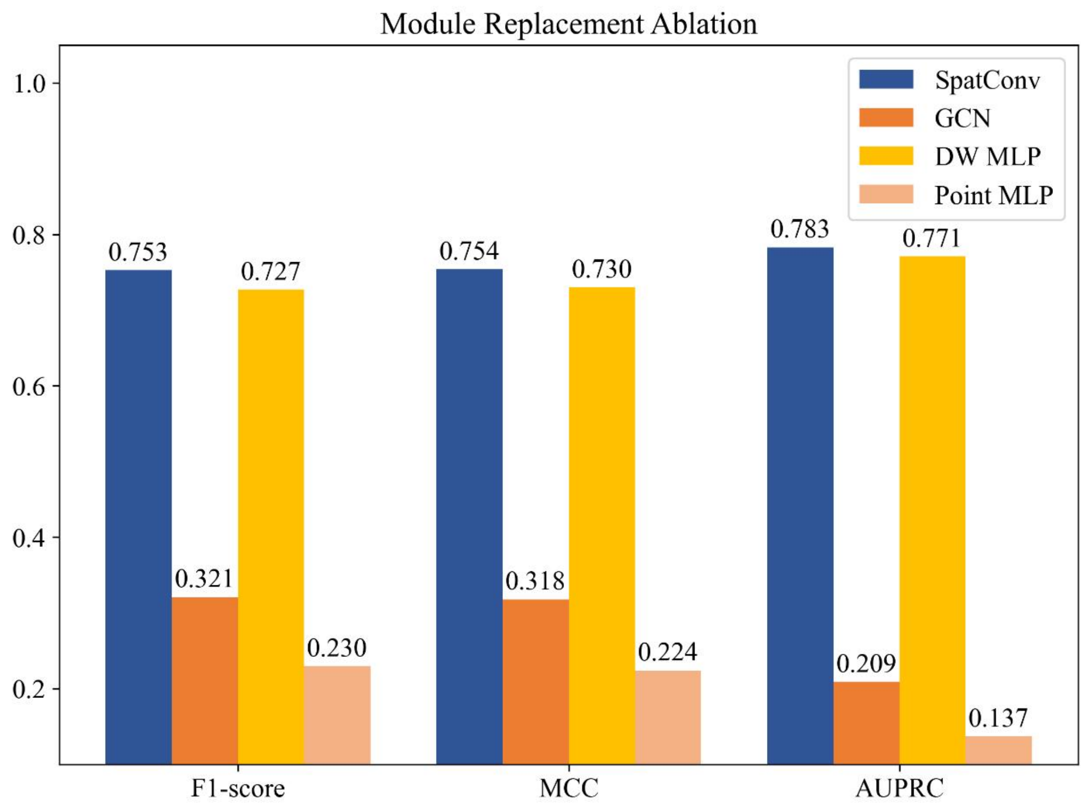

Figure S9 Module replacement ablation study of the feature aggregation component on ZN^{2+} data.

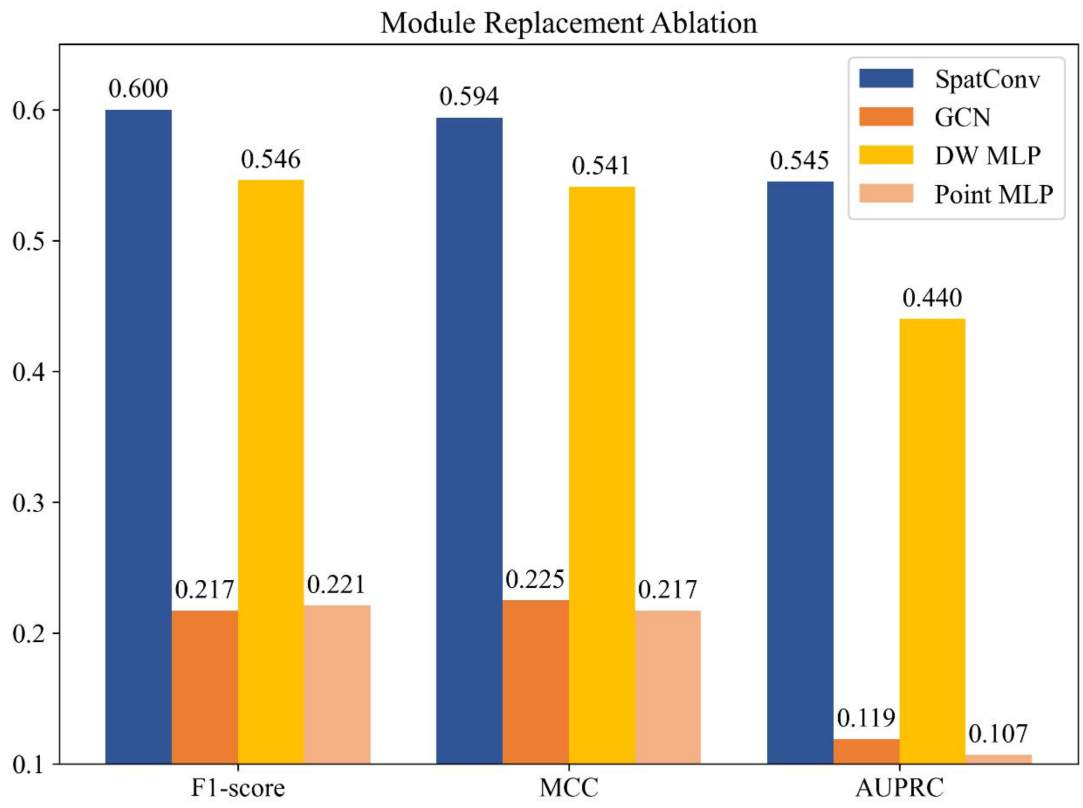

Figure S10 Module replacement ablation study of the feature aggregation component on Mn^{2+} data.

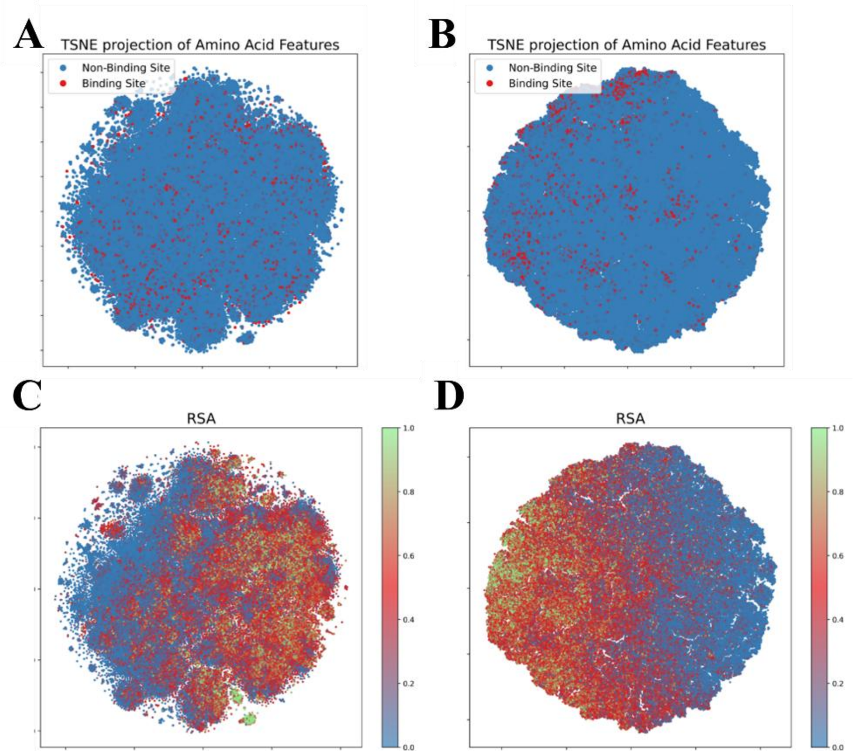

Figure S11 t-SNE projections of peptide binding site residue feature before and after training.

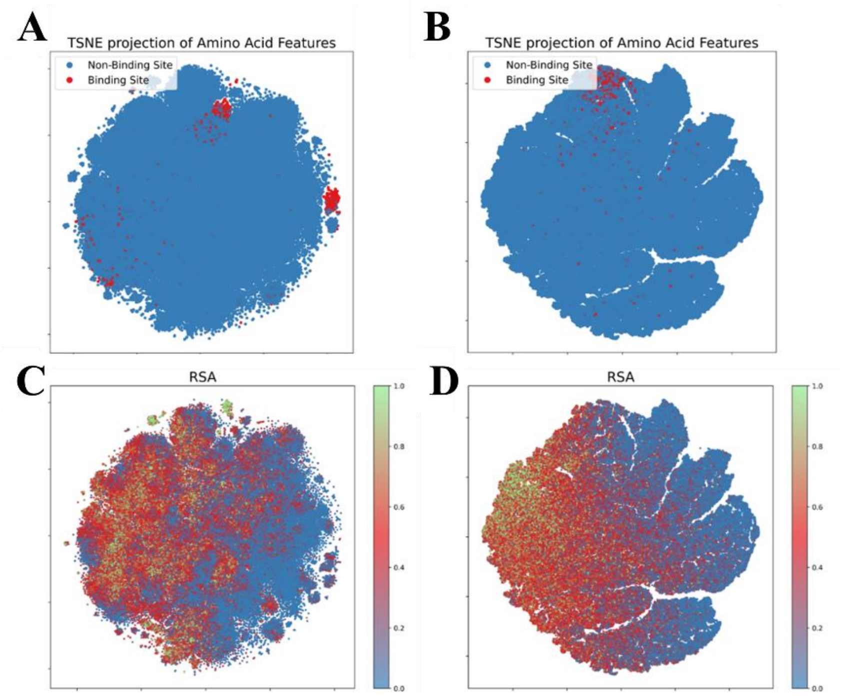

Figure S12 t-SNE projections of Zn^{2+} binding site residue feature before and after training.

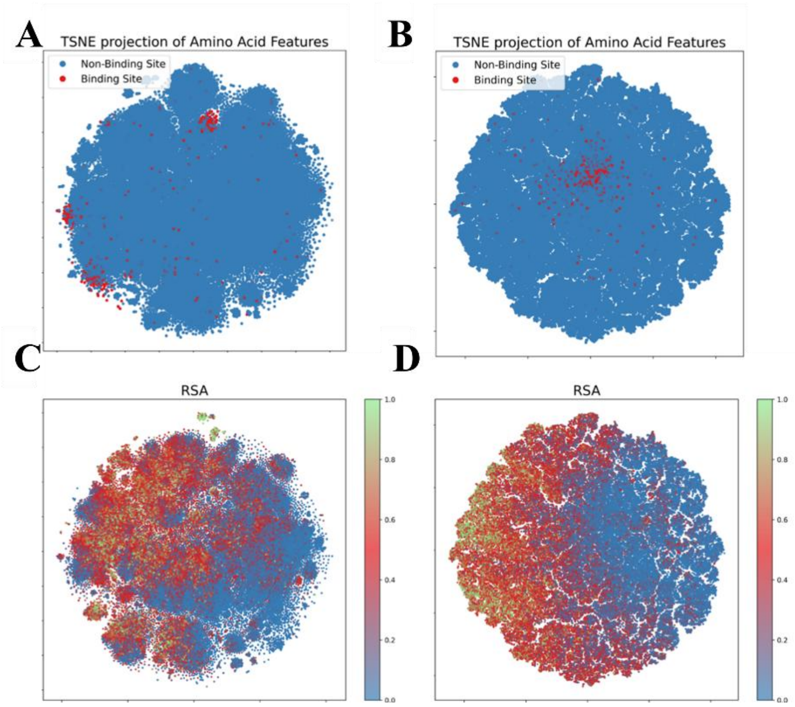

Figure S13 t-SNE projections of Mn^{2+} binding site residue feature before and after training.

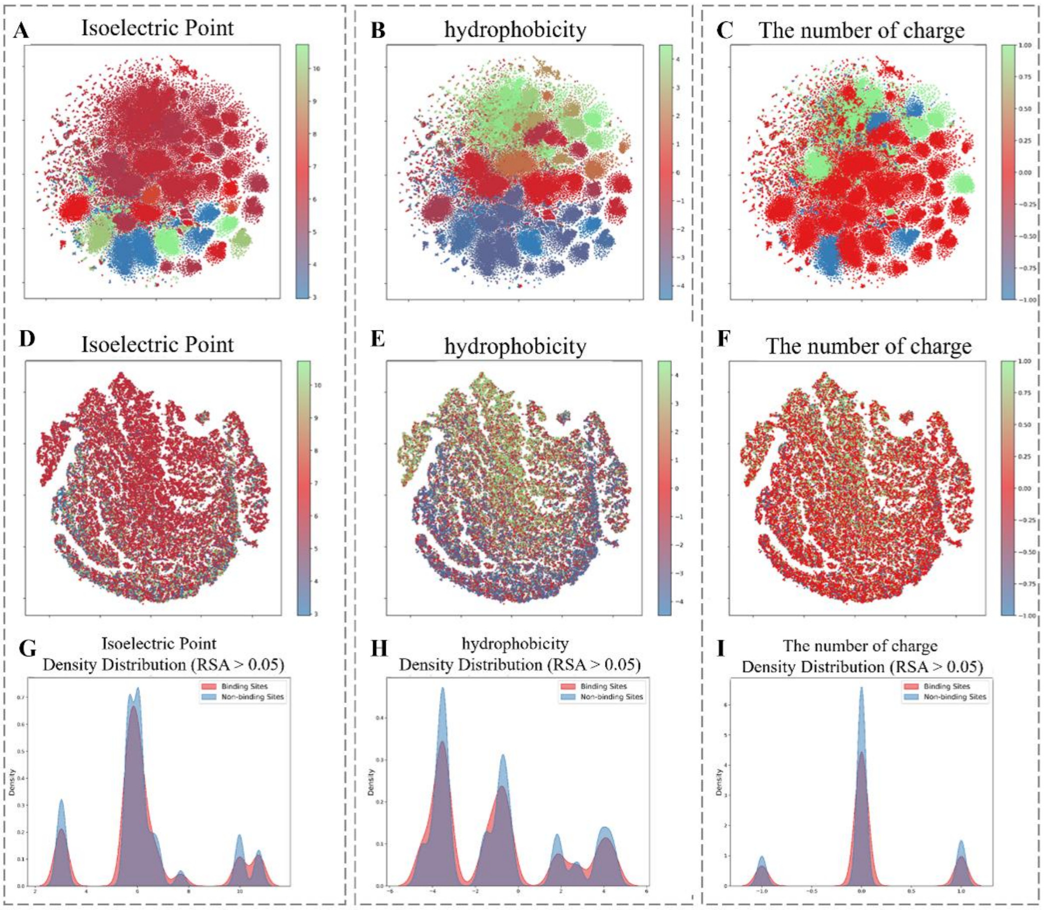

Figure S14 t-SNE visualization of protein physicochemical properties in initial and SpatConv trained states, alongside density plots of physicochemical property distributions for binding/non-binding sites. a-c, Respectively, display the distribution of

the initial isoelectric point, hydrophobicity, and number of charge features. **d-f,** Show the corresponding feature distributions after model training. **g-i,** Respectively show the density distributions of isoelectric point, hydrophobicity, and number of charge features for both binding sites (red) and non-binding sites (blue) with RSA > 0.05.

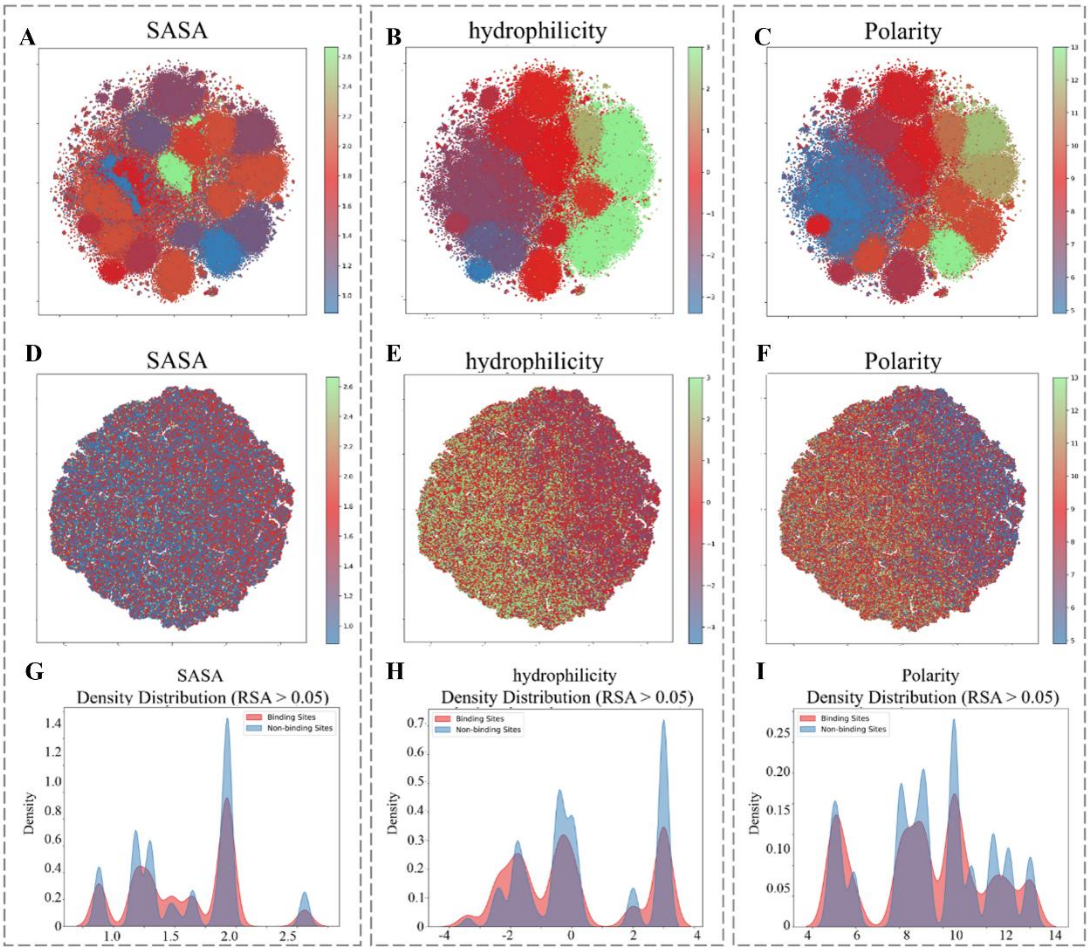

Figure S15 t-SNE visualization of the physicochemical properties of proteins binding with peptides in initial and SpatConv trained states, alongside density plots of physicochemical property distributions for binding/non-binding sites.

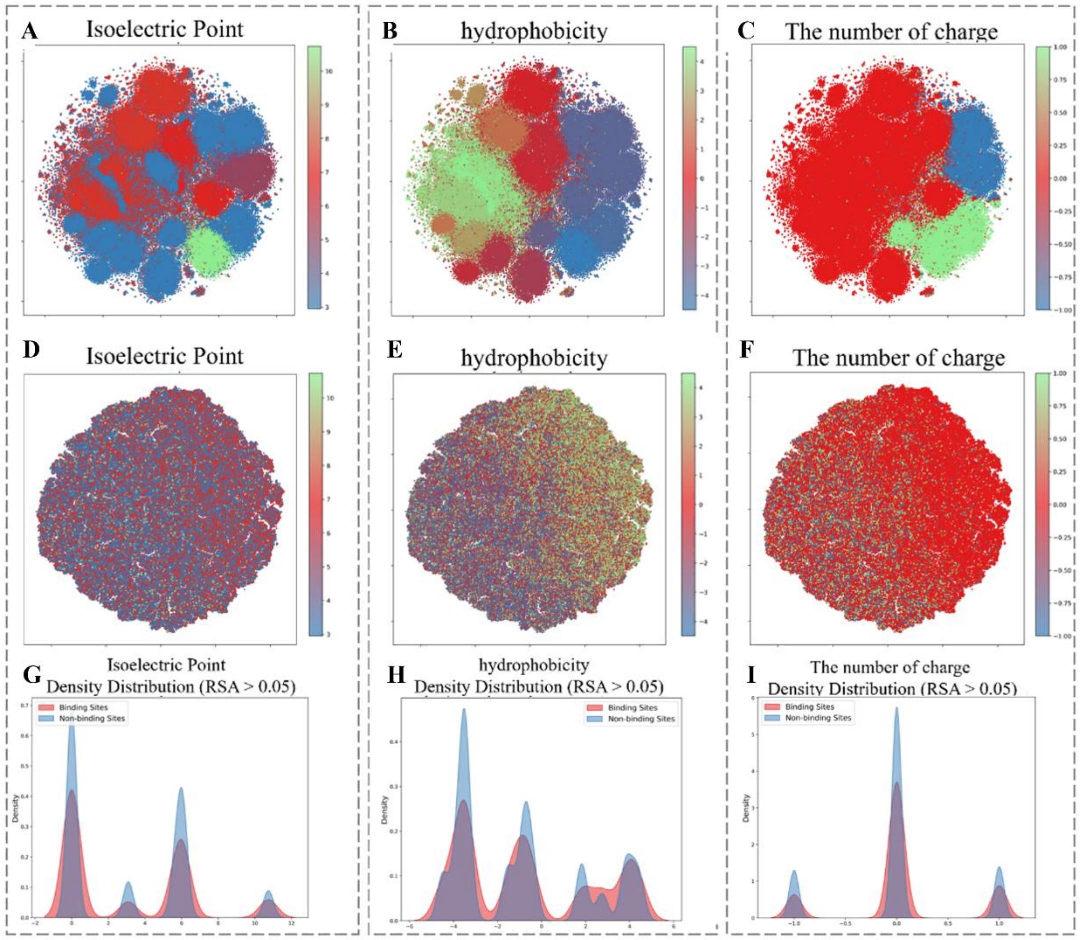

Figure S16 t-SNE visualization of the physicochemical properties of proteins binding with peptides in initial and SpatConv trained states, alongside density plots of physicochemical property distributions for binding/non-binding sites.

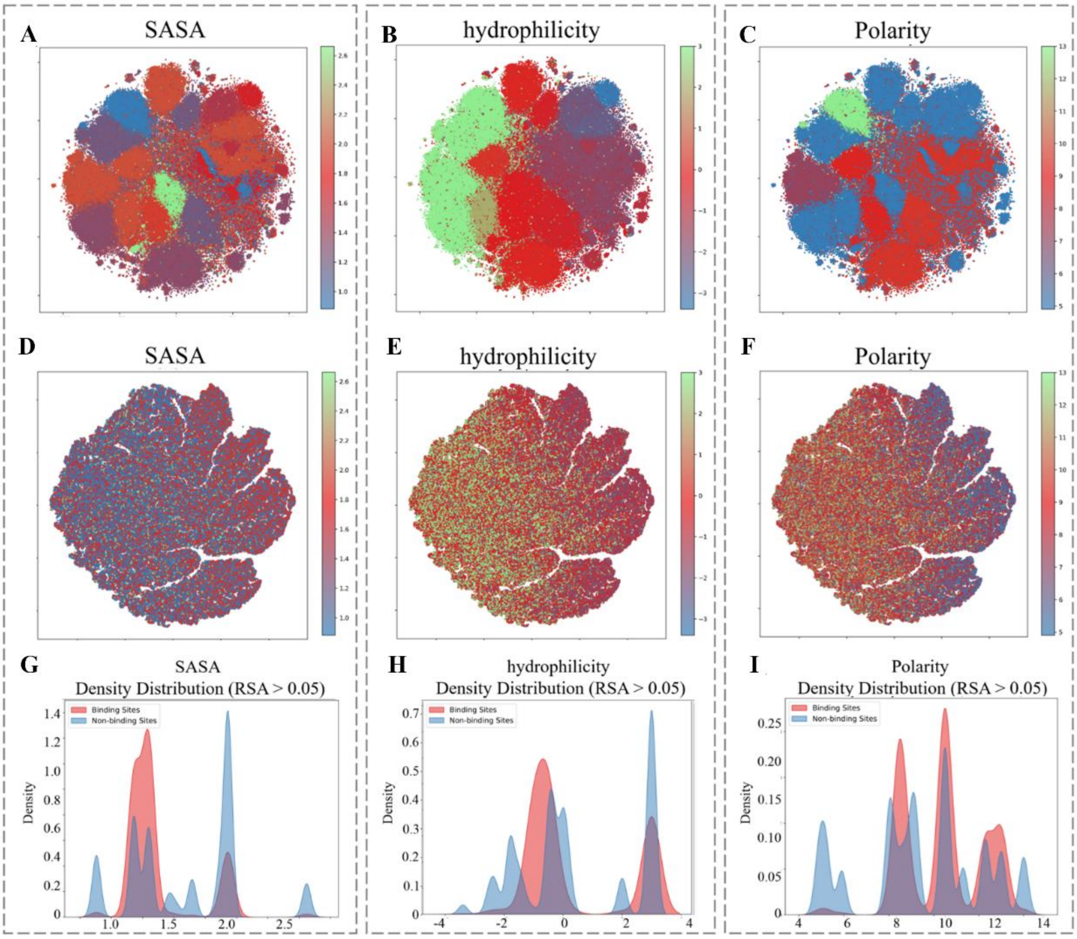

Figure S17 t-SNE visualization of the physicochemical properties of proteins binding with Zn^{2+} in initial and SpatConv trained states, alongside density plots of physicochemical property distributions for binding/non-binding sites.

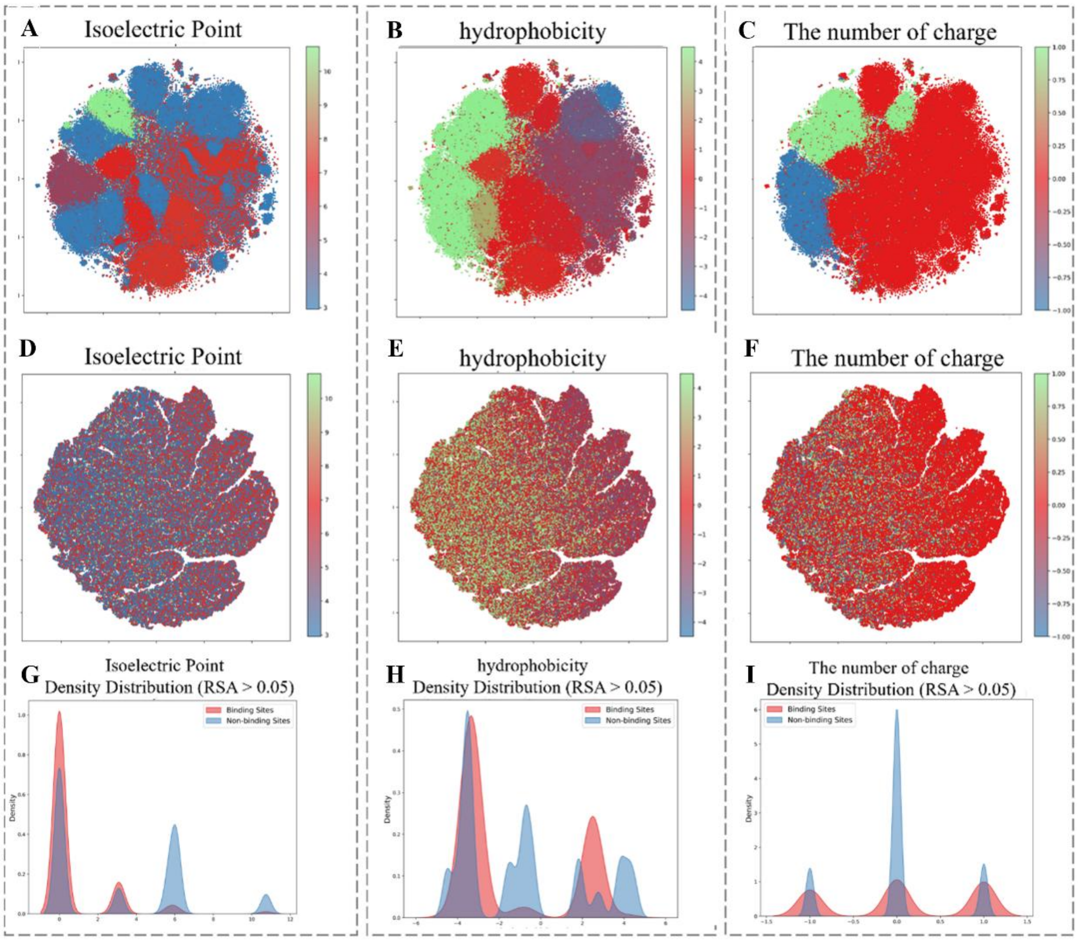

Figure S18 t-SNE visualization of the physicochemical properties of proteins binding with Zn^{2+} in initial and SpatConv trained states, alongside density plots of physicochemical property distributions for binding/non-binding sites.

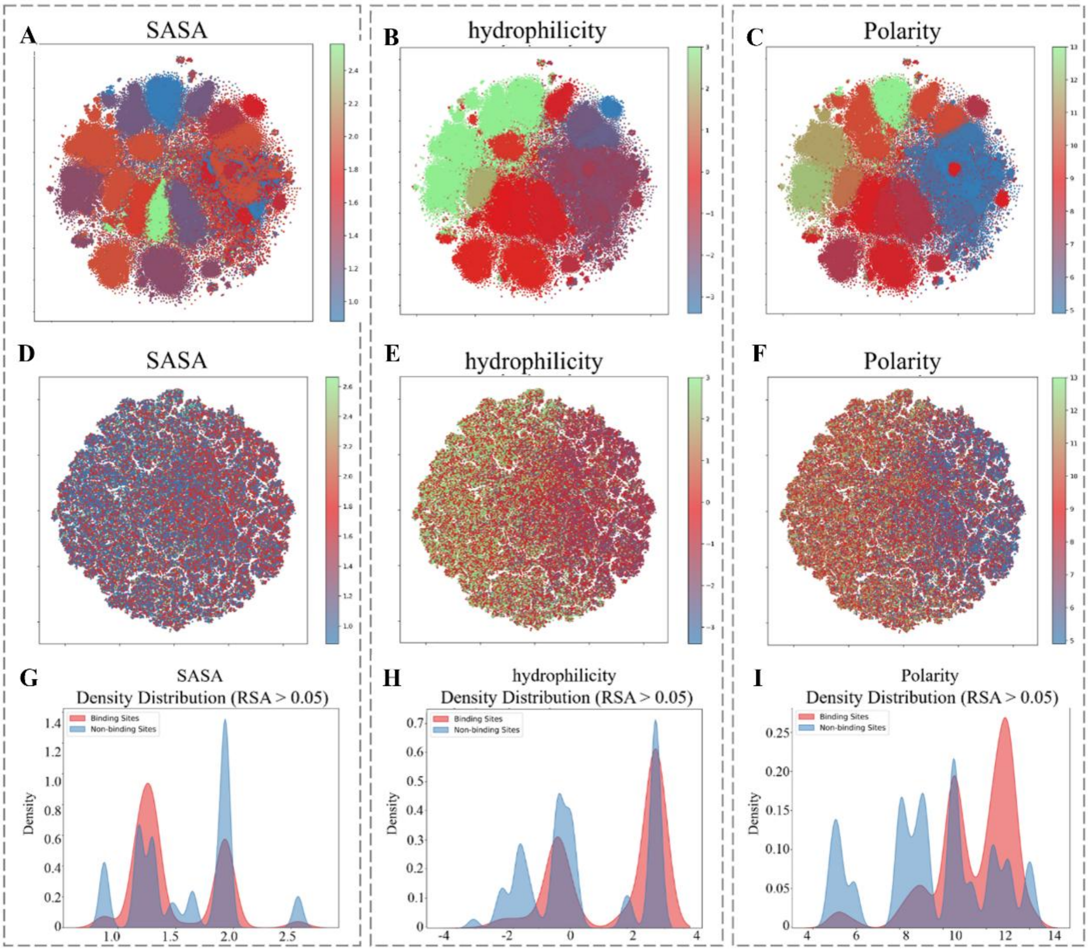

Figure S19 t-SNE visualization of the physicochemical properties of proteins binding with Mn^{2+} in initial and SpatConv trained states, alongside density plots of physicochemical property distributions for binding/non-binding sites.

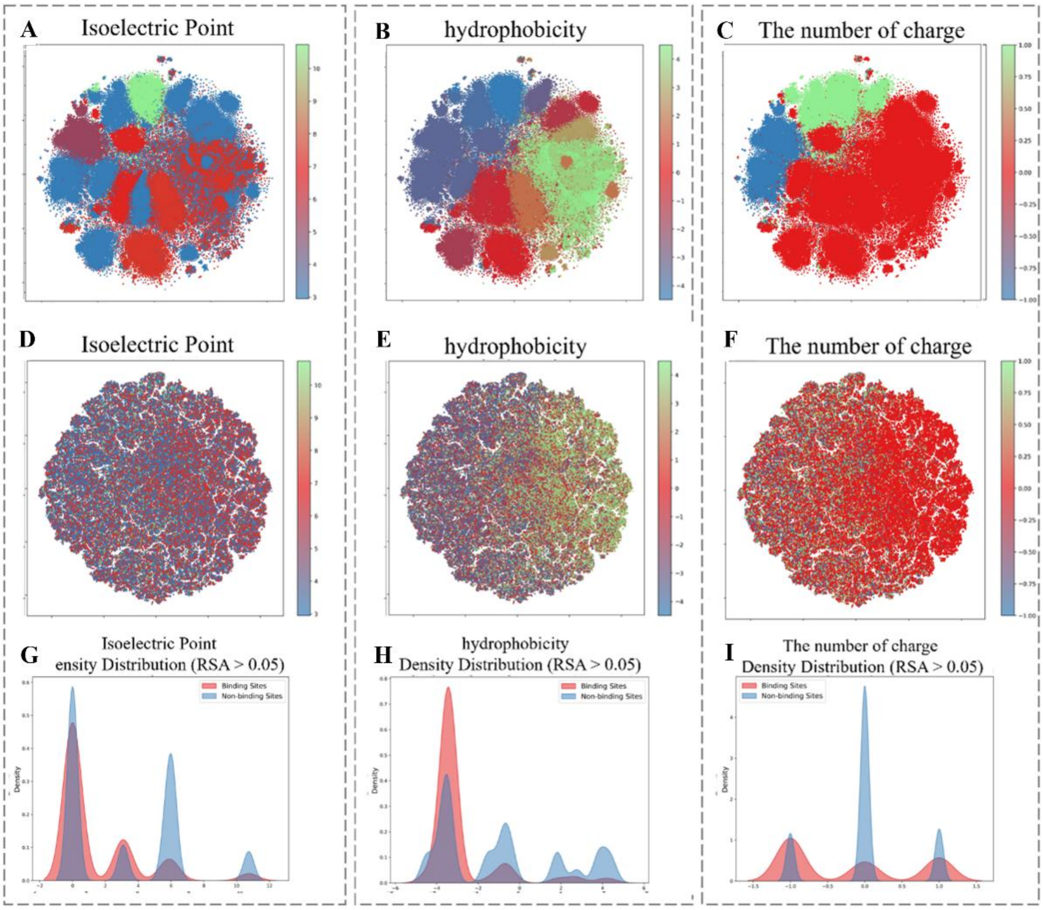

Figure S20 t-SNE visualization of the physicochemical properties of proteins binding with Mn^{2+} in initial and SpatConv trained states, alongside density plots of physicochemical property distributions for binding/non-binding sites.

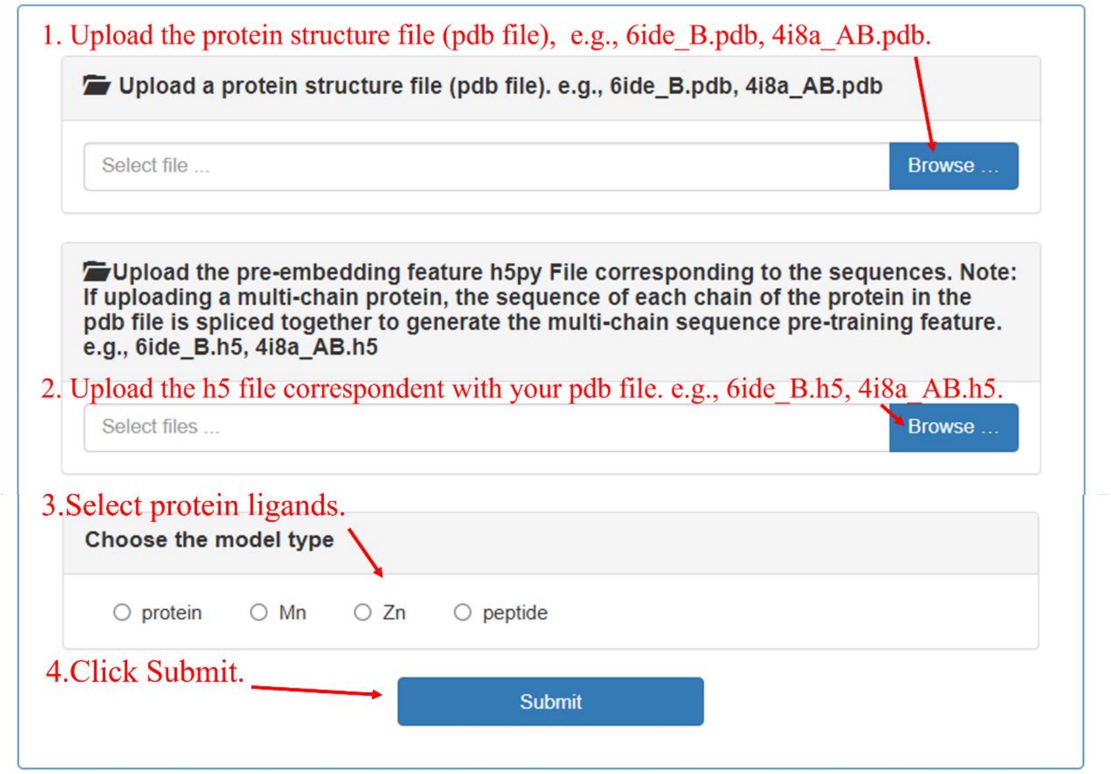

Figure S21 The interface of the SpatConv web server.

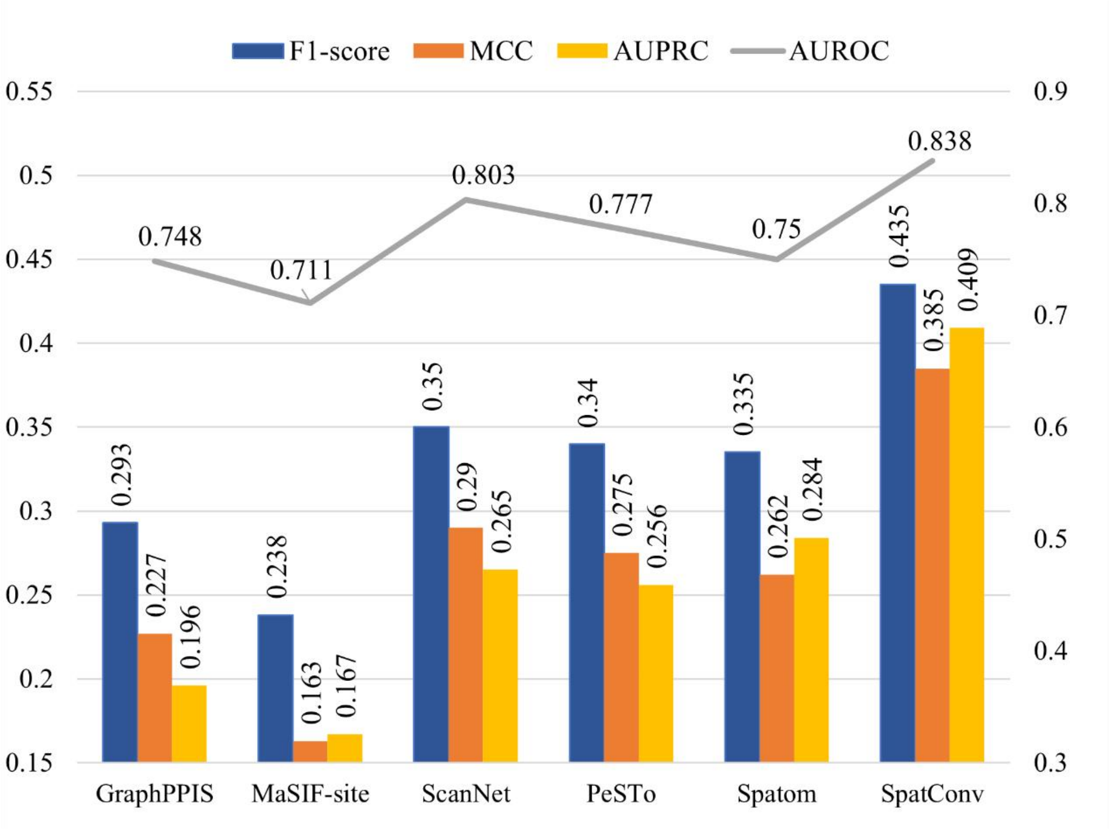

Figure S22. Performance of SpatConv and other compared methods on new Test set.

References

1. Wrapp, D. et al. Cryo-EM structure of the 2019-nCoV spike in the prefusion conformation. Science 367, 1260-1263, doi:doi:10.1126/science.abb2507 (2020).
2. Walls, A. C. et al. Structure, Function, and Antigenicity of the SARS-CoV-2 Spike Glycoprotein. Cell 181, 281-292.e286, doi:https://doi.org/10.1016/j.cell.2020.02.058 (2020).
3. Yang, Y., Li, F. & Du, L. Therapeutic nanobodies against SARS-CoV-2 and other pathogenic human coronaviruses. J Nanobiotechnol 22, 304, doi:10.1186/s12951-024-02573-7 (2024).
4. Cerutti, G. et al. Potent SARS-CoV-2 neutralizing antibodies directed against spike N-terminal domain target a single supersite. Cell Host & Microbe 29, 819-833.e817, doi: 10.1016/j.chom.2021.03.005 (2021).
5. Harris, C. R. et al. Array programming with numpy. Nature 585, 357 (2020).
6. Chollet, F. Deep Learning with Python (Simon and Schuster, 2017).
7. Paszke, A., Gross, S., Massa, F., Lerer, A., Bradbury, J., Chanan, G., Killeen, T., Lin, Z., Gimelshein, N., Antiga, L., Desmaison, A., Kopf, A., Yang, E., DeVito, Z., Raison, M., Tejani, A., Chilamkurthy, S., Steiner, B., Fang, L., Bai, J., & Chintala, S. PyTorch: An Imperative Style, High-Performance Deep Learning Library. Advances in Neural Information Processing Systems 32 (NeurIPS 2019), 8024-8035 (2019).
8. Cock, P. J. et al. Biopython: freely available python tools for computational molecular biology and bioinformatics. Bioinformatics 25, 1422 (2009).
9. Lam, S. K., Pitrou, A. & Seibert, S. Numba: a LLVM-based Python JIT compiler, in Proc. Second Workshop on the LLVM Compiler Infrastructure in HPC 1–6. Venue: Austin, TX, USA. Editor: H. Finkel (2015).
10. Virtanen, P. et al. Scipy 1.0: fundamental algorithms for scientific computing in python. Nat. Methods 17, 261 (2020).
11. Pedregosa, F. et al. Scikit-learn: machine learning in python. J. Mach. Learning Res. 12, 2825 (2011).
